# Oxidative stress monitoring in iPSC-derived motor neurons using genetically encoded biosensors of H_2_O_2_

**DOI:** 10.1038/s41598-022-12807-z

**Published:** 2022-05-27

**Authors:** Elizaveta Ustyantseva, Sophia V. Pavlova, Anastasia A. Malakhova, Kirill Ustyantsev, Suren M. Zakian, Sergey P. Medvedev

**Affiliations:** 1 The Federal Research Center Institute of Cytology and Genetics, Siberian Branch of Russian Academy of Sciences, 10, Lavrentiev Ave, 630090 Novosibirsk, Russia; 2 Institute of Chemical Biology and Fundamental Medicine, Siberian Branch of the Russian Academy of Sciences, 8, Lavrentiev Ave., 630090 Novosibirsk, Russia; 3 E. Meshalkin National Medical Research Center of the Ministry of Health of the Russian Federation, 15 Rechkunovskaya Str., 630055 Novosibirsk, Russia

**Keywords:** Neurological models, Genetic engineering, Cell culture, Mechanisms of disease, Cellular neuroscience, Diseases of the nervous system

## Abstract

Oxidative stress plays an important role in the development of neurodegenerative diseases, being either the initiator or part of a pathological cascade that leads to the neuron’s death. Genetically encoded biosensors of oxidative stress demonstrated their general functionality and overall safety in various systems. However, there is still insufficient data regarding their use in the research of disease-related phenotypes in relevant model systems, such as human cells. Here, we establish an approach for monitoring the redox state of live motor neurons with *SOD1* mutations associated with amyotrophic lateral sclerosis. Using CRISPR/Cas9, we insert genetically encoded biosensors of cytoplasmic and mitochondrial H_2_O_2_ in the genome of induced pluripotent stem cell (iPSC) lines. We demonstrate that the biosensors remain functional in motor neurons derived from these iPSCs and reflect the differences in the stationary redox state of the neurons with different genotypes. Moreover, we show that the biosensors respond to alterations in motor neuron oxidation caused by either environmental changes or cellular stress. Thus, the obtained platform is suitable for cell-based research of neurodegenerative mechanisms.

## Introduction

Redox reactions are part of cellular metabolism. Generally, reactive oxygen species (ROS), emerging as a byproduct of such reactions, are quickly neutralized by antioxidant systems^1^. In oxidative stress, ROS accumulate due to excessive production or disturbed utilization, leading to cell malfunction^2^. An increasing number of ROS molecules alters protein structure, changes properties of membranes due to lipid peroxidation, and causes DNA damage, which makes oxidative stress one of the major mechanisms of degenerative disorders and aging^3,4^.

Oxidative stress is known to play an important role in various pathologies, and its involvement in the development of neurodegenerative diseases is undeniable, although not always clear^5,6^. Indeed, oxidative stress is strongly associated with amyotrophic lateral sclerosis (ALS)—a disorder characterized by the inevitable death of motor neurons (MNs) leading to progressive paralysis^7^. The first ALS-associated gene, *SOD1*, encoding the antioxidant protein superoxide dismutase 1, was discovered in 1993; thus, oxidative stress has been proposed as the primary pathological mechanism of the disease^8^. However, subsequent studies revealed that ALS has a much more complex etiology involving other genes and that only 10% of cases are hereditary^9–11^. Nonetheless, signs of oxidative damage have been found in both patients and model organisms, regardless of the initial cause of the disease, suggesting a universal role of redox imbalance in MN damage^12–14^.

Redox studies are often conducted by measuring key molecules, including but not limited to glutathione (GSH/GSSG), hydrogen peroxide (H_2_O_2_), and NADP^+^/NADPH^15,16^. Although dozens of molecular chemical probes have been developed for such analyses, most have low specificity and availability and interfere with cellular processes^17,18^. Genetically encoded biosensors can be applied for the same measurement as molecular probes^19^. These protein-based detectors are initially delivered inside the cell in the form of nucleic acid. The cell subsequently produces biosensor molecules as long as the coding sequence is available. Since the biosensor molecules are produced inside the cell, they are not limited by availability and, therefore, can be applied not only in cell culture but also in more complex model systems, such as animals or plants^20–22^. Furthermore, the nature of genetically encoded biosensors allows for easy modifications, such as the addition of signal peptides that direct the biosensor to specific cellular compartments (nucleus, mitochondria, endoplasmic reticulum, and plasma membrane)^23,24^. Traditional methods for research with genetically encoded biosensors in cell culture imply transient expression via plasmid delivery or viral-mediated integration of the biosensors sequences^4^. The first approach provides a high-intensity signal but does not allow long-term experiments since the plasmid copy number reduces by half with every cell division. The second method, by contrast, provides a stable expression of the biosensor but does not guarantee reliable results since randomly integrated biosensors can disturb genome function^25^. Although many redox biosensors have been developed in recent years, only a few have been validated in model systems as suitable for studying disease-associated phenotypes^20,26–28^.

Here, we developed a platform for monitoring oxidative stress in live MNs using reduction–oxidation sensitive green fluorescent protein 2 (roGFP2)-based biosensors differentially targeted to the cytoplasm (Cyto-roGFP2-Orp1) and mitochondria (Mito-roGFP2-Orp1). These are well-known ratiometric biosensors that reflect H_2_O_2_ level, hence, ***relative*** oxidation in the respective cellular compartments^29^. First, to create a basis for our research, we generated isogenic induced pluripotent stem cell (iPSC) lines with mutations affecting different parts of the ALS-associated *SOD1* gene. Next, we allocated the biosensors sequences in the “safe harbor” *AAVS1* locus of these iPSC lines under the control of the Tet-On doxycycline-inducible expression system. This approach allowed us not only to avoid the potential adverse effect of the insert but also to generate cell lines with a stable yet controllable expression of the biosensors. Finally, we assessed the functionality of the biosensors in MNs, derived from the generated iPSCs, and showed that they reflect changes in the oxidation of these cells in conditions of direct increase of ROS in the cellular environment, as well as during neuronal-specific glutamate-induced stress. Thus, this platform can serve as an adequate model to study redox-associated pathologies in neurodegenerative diseases.

## Results

### Introduction of the *SOD1* D91A and G128R mutations in iPSCs of the clinically healthy donor

**Figure 1:**
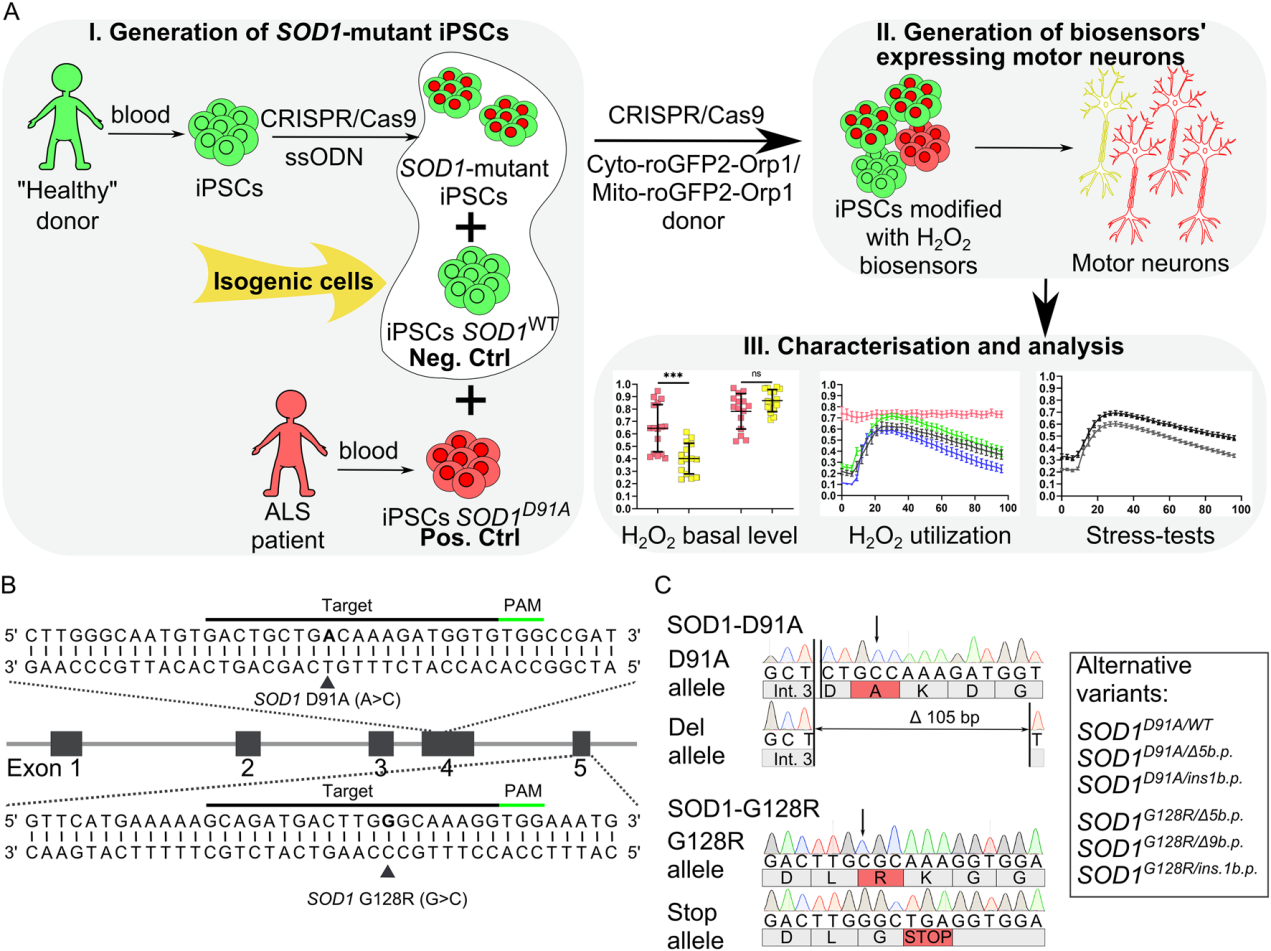
Generation of isogenic *SOD1*-mutant iPSC lines. (**A**) Schematic representation of the experimental design. Induced pluripotent stem cell (iPSC) lines with *SOD1* mutations were generated either from iPSCs of a healthy donor by CRISPR/Cas9-mediated genome editing or obtained from a patient with ALS. The iPSCs from a healthy donor served as an isogenic negative control (Neg. Ctrl); patient-specific iPSCs served as a positive control (Pos. Ctrl). Next, the iPSC lines (control, *SOD1*-mutated, and patient-specific) were modified with biosensors of cytoplasmic (Cyto-roGFP2-Orp1) and mitochondrial (Mito-roGFP2-Orp1) H_2_O_2_ by targeted insertion of the biosensors’ sequences with CRISPR/Cas9. Then, the modified iPSC lines were differentiated in spinal motor neurons with subsequent analysis of the redox state of the neurons with the biosensors. (**B**) Schematic of the *SOD1* gene with partial sequences of exons 4 and 5. Protospacers designed for CRISPR/Cas9-mediated double-stranded breaks are underlined with black lines, protospacer adjacent motif (PAM)—with green lines; target mutations are in bold and marked with black triangles. (**C**) Partial *SOD1* sequences of exons 4 and 5 of the SOD1-D91A and SOD1-G128R iPSC lines. Int. 3—intron 3, substituted nucleotides marked with an arrow, corresponding mutated amino acids are highlighted in red. The box contains a list of clones with alternative *SOD1* variants obtained in the study.

The *SOD1* gene has more than 140 mutations associated with ALS, which define the clinical features of the disease such as its manifestation age, rate of progression, presence of additional symptoms, etc.^30^. We chose c.272A>C and c.382G>C mutations that lead to either a relatively benign or severe disease course, respectively^31,32^. In the first step of our workflow (Fig. 1A), we designed corresponding CRISPR/Cas9 guide RNA targeting the sequences in the exons 4 and 5 of *SOD1* and single-stranded oligodeoxynucleotides (ssODN) donor templates necessary for the introduction of c.272A>C and c.382G>C single-nucleotide mutations that lead to the D91A and G128R substitutions, respectively, in the SOD1 polypeptide (Fig. 1B, Supplementary Table S1).

We introduced these mutations, using CRISPR/Cas9, into a well-characterized control iPSC line (K7-4Lf), obtained earlier from a clinically healthy individual^33^ (Supplementary Table S2) and recovered 66 clones for the D91A variant and 124 clones for the G128R variant of which 6 (9.1%) and 4 (3.2%) clones, respectively, were positive for the target mutations. As a result, several clones with different *SOD1* allelic variants were obtained (Fig. 1C). Since we did not find any homozygous variants, we chose clones with *SOD1*^*D91*^^*A/del105*^ (iPSC line SOD1-D91A) and *SOD1*^G128R/K129*^ (iPSC line SOD1-G128R) variants for subsequent experiments (Supplementary Fig. S3). Importantly, these clones showed no mutations in five of the most likely predicted off-target sites (Supplementary Fig. S4).

### Generation of iPSC lines modified with genetically encoded biosensors of H_2_O_2_ via CRISPR/Cas9-mediated *AAVS1* targeting

We introduced sequences of two genetically encoded biosensors, Cyto-roGFP2-Orp1, and Mito-roGFP2-Orp1, measuring H_2_O_2_ levels in the cytoplasm and mitochondria respectively, in the genome of SOD1-D91A, SOD1-G128R, and the isogenic control line (K7-4Lf) to obtain stable expression. Additionally, we introduced the same sequences in the genome of a patient-specific iPSC line (iALS) previously generated in our lab from a person diagnosed with a hereditary form of ALS with a homozygous D91A mutation in *SOD1*^34^.

**Figure 2:**
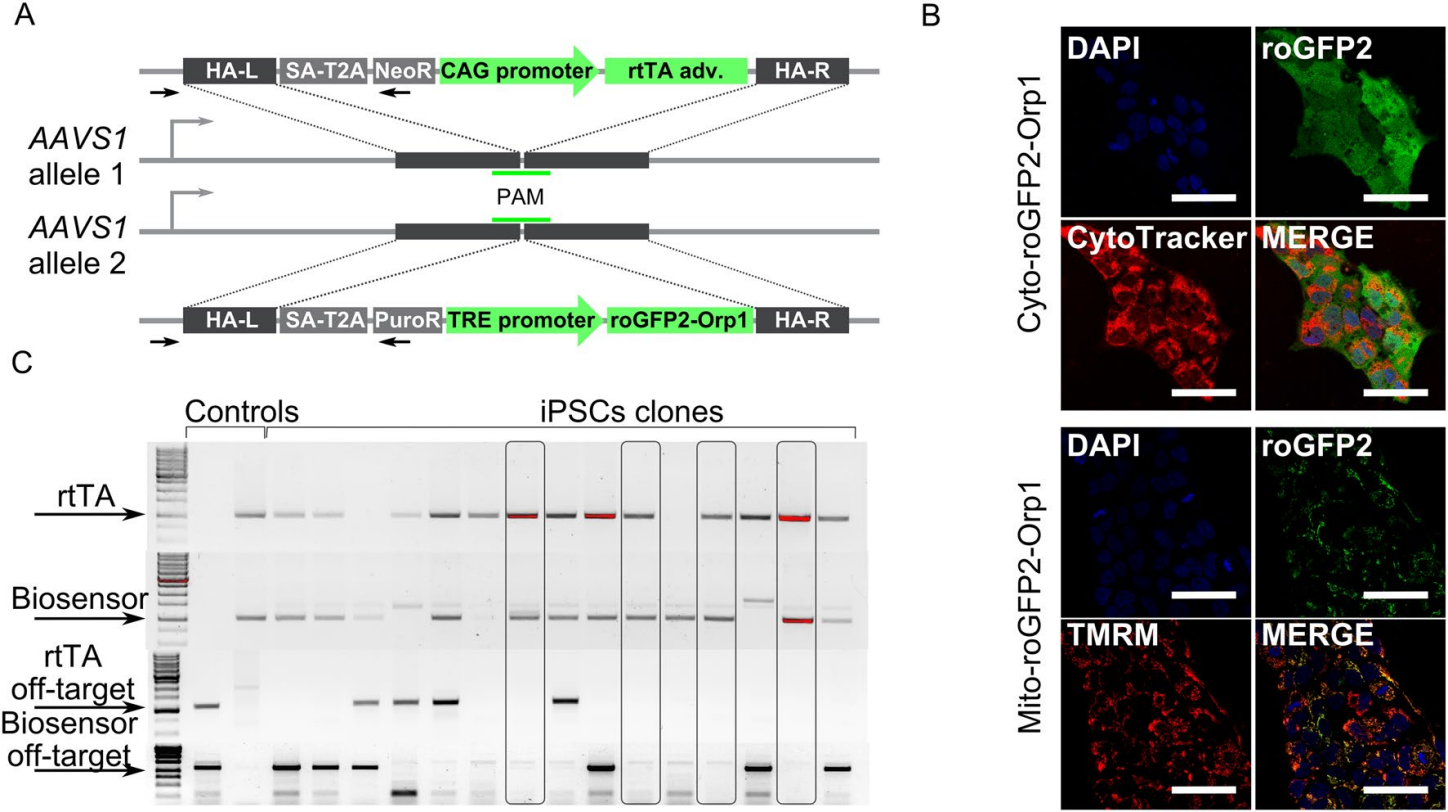
Generation of iPSCs expressing the Cyto-roGFP2-Orp1 and Mito-roGFP2-Orp1 biosensors. (**A**) Schematic of the biosensor and transactivator for doxycycline-controllable expression inserts in the *AAVS1* locus. In one allele, homologous arms (HA-L—left, HA-R—right) flank the splice acceptor site (SA), T2A peptide, neomycin-resistance gene, and reverse tetracycline-controlled transcriptional activator (rtTA) under control of the CAG promoter. In another allele, homologous arms flank the splice acceptor site (SA), T2A peptide, puromycin-resistance gene, and the biosensor’s sequence under control of the TRE-promoter. The primers used to detect the target insertions are represented as black arrows. (**B**) Representative images of live iPSCs expressing the Cyto-roGFP2-Orp1 (top) or Mito-roGFP2-Orp1 (bottom) biosensors 24 h after the addition of 2 mg/ml doxycycline in the medium. Scale bar 50 μm. TMRM—tetramethylrhodamine methyl ester perchlorate. (**C**) Screening of the iPSC clones for the target (two top gels) and off-target (two bottom gels) inserts. The arrows mark target PCR products. The clones positive for the target and negative for the off-target inserts are in frames.

The Tet-On system, applied for the biosensors expression, consists of two elements: the biosensor's sequence under the control of the tetracycline-dependent promoter and the specific transactivator (rtTA, reverse tetracycline-controlled transactivator) essential for the controlled expression of the target genes^35^. To deliver these elements in the cell’s genome, we used biallelic target insertion in the safe harbor *AAVS1* locus via CRISPR/Cas9 (Fig. 2A). The donor plasmids were either obtained from the vendor or constructed in our laboratory^36,37^ (Supplementary Fig. S5). IPSC clones with the target insertions were selected using respective media and tested for the presence of the biosensors’ expression in response to doxycycline (tetracycline derivative) addition (Fig. 2B). Selected clones were further examined for target and off-target insertions by PCR (Fig. 2C). Three clones for each cell line were selected for differentiation and analysis (Supplementary Fig. S6).

### Neuronal differentiation of the modified iPSC lines and the biosensor expression in iPSC-derived motor neurons

**Figure 3:**
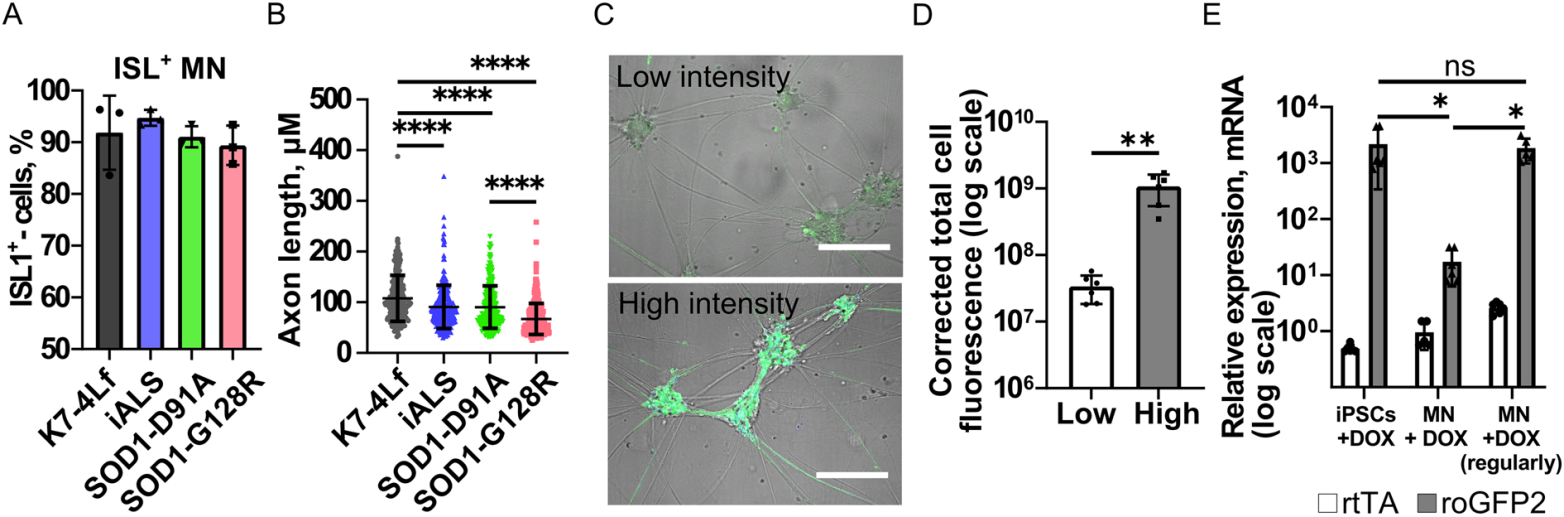
Generation of iPSC-derived motor neurons (MNs) expressing the Cyto-roGFP2-Orp1 and Mito-roGFP2-Orp1 biosensors. (**A**) Quantification of flow cytometry analysis of ISL-positive cells was performed on day 20 of the differentiation protocol: K7-4Lf (isogenic control MNs without mutations), iALS (patient-specific MNs with D91A mutation in *SOD1*), SOD1-D91A (isogenic MNs with D91A mutation in *SOD1*), and SOD1-G128R (isogenic MNs with G128R mutation in *SOD1*). Data (N = 3 independent differentiations) are the mean ± standard deviation (S.D.) (**B**) Axon length quantification (differentiation day 21). Data was collected from three independent differentiations with a total of 257, 270, 409, and 397 cells analyzed for K7-4Lf, iALS, SOD1-D91A, and SOD1-G128R MNs, respectively, and are presented as the mean ± S.D. (**C**) Representative images of the MNs with low (top) and high (bottom) intensity signals form Cyto-roGFP2-Orp1. Scale bar 100 μm. (**D**) Quantification of the Cyto-roGFP2-Orp1 fluorescence intensity in the MNs with low and high Cyto-roGFP2-Orp1 signal intensity. Data (N = 6 independent differentiations) are the mean ± S.D. (**E**) RT-qPCR analysis of the tetracycline-controlled transcriptional activator (rtTA) and biosensors (roGFP2) mRNA expression 48 h after 2 μg/ml doxycycline addition. iPSCs + DOX—iPSCs expressing biosensor; MN + DOX—MNs supplemented with doxycycline after completion of the differentiation protocol; MN + DOX (regularly)—MNs differentiated with regular doxycycline supplementation. Data (N = 6 independent differentiation) are the mean ± S.D. **p < 0.01, ****p < 0.0001, one-way ANOVA with post hoc Tukey’s tests for (**B**) and (**E**), Welch t-test for (**D**).

We utilized a previously described protocol of highly efficient MN differentiation^38^. All iPSC-derived MNs stained positively for the most common markers: choline acetyltransferase (ChAT), ISL LIM homeobox 1 (ISL1), and motor neuron and pancreas homeobox (MNX1), and expressed mRNA of these proteins (Supplementary Fig. S7). Differentiation efficiency, analyzed on day 20 of differentiation by counting the ISL-positive cells using flow cytometry, showed 89–95% of MNs in the samples (Fig. 3A).

To characterize MNs obtained, we measured the axonal length of the iPSC-derived MNs on day 21 of differentiation. SOD1-D91A (90.7 ± 42.6 μm) and iALS (90.4 ± 41.9 μm) MNs had considerably shorter axonal processes compared to the control K7-4Lf MNs (107.8 ± 45.5 μm). The mean axon length in SOD1-G128R (67 ± 30.6 μm) MNs was even lower, suggesting a highly disturbed function of these cells (Fig. 3B).

The *AAVS1* site is located in the intron of a transcriptionally active gene and was described previously as suitable for stable expression of transgenes^39^. However, we have discovered that the MNs did not always retain a detectable fluorescence level of the biosensor at the terminal stages of differentiation, and this did not depend on a particular cell line (Fig. 3C,D). Analysis of the expression level of rtTA and roGFP2 in the MNs with low intensity of the biosensor signal revealed that the terminally differentiated MNs expressed mRNA of the rtTA at the same level as the corresponding iPSCs from which they were obtained. At the same time, the expression of the biosensor roGFP2 was decreased by two orders, suggesting that the promoter of the biosensor was selectively inhibited (Fig. 3E). We performed differentiation, supplementing the medium regularly with doxycycline from the first day of differentiation to keep the biosensor promoter in an active state. As a result, the biosensor retained high signal intensity in the differentiated MNs as well as mRNA expression on a level comparable to the iPSCs (Fig. 3E). Moreover, the dynamic range (difference between fully oxidized and fully reduced biosensor’s state) of the signals generated in the MNs was relatively stable across the lines, suggesting a similar level of the biosensor expression (Supplementary Fig S8).

### Nutrient deprivation affects the mitochondrial level of H_2_O_2_ regardless of the genotype

**Figure 4:**
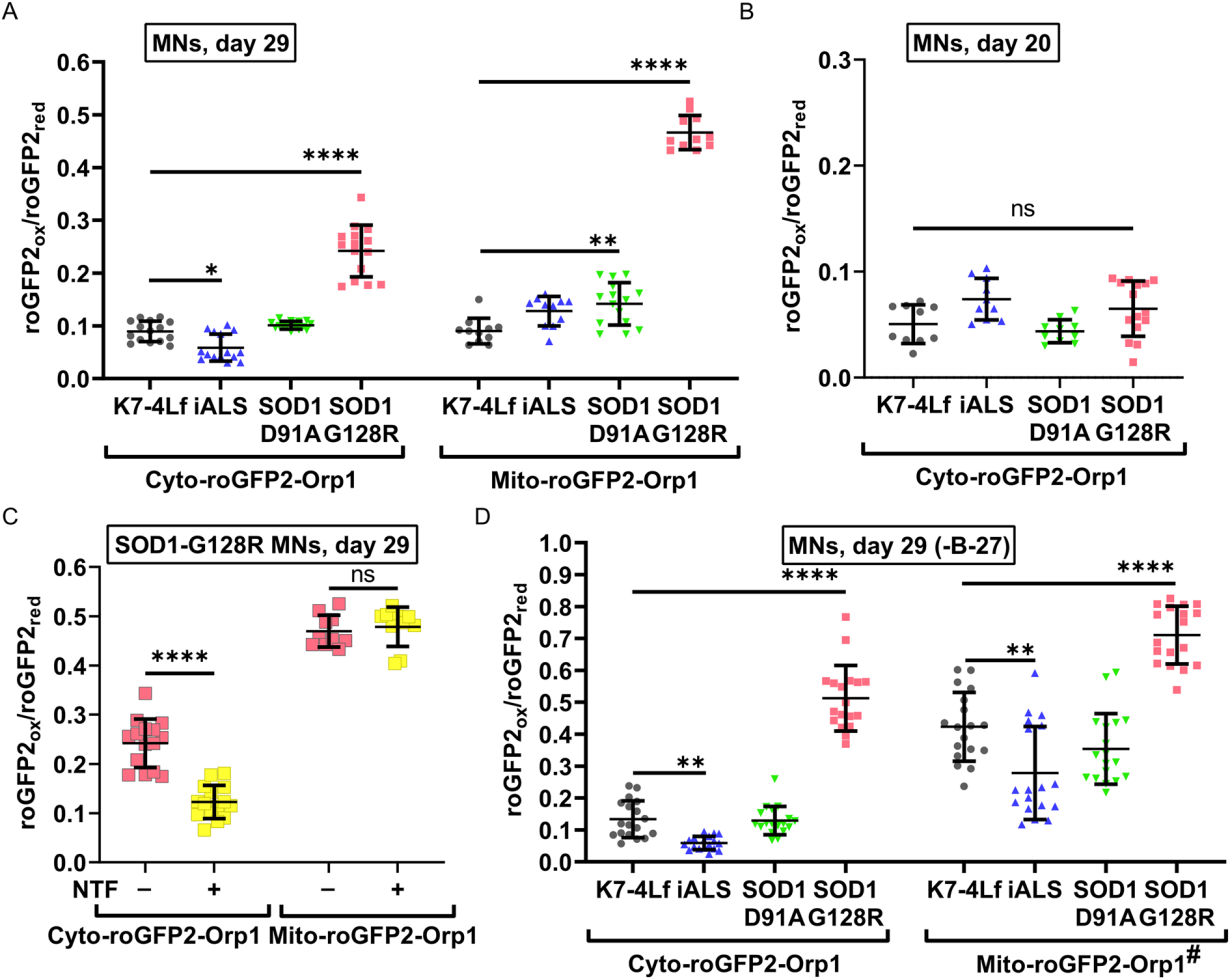
Basal levels of H_2_O_2_ in the cytoplasm (Cyto-roGFP2-Orp1) and mitochondria (Mito-roGFP2-Orp1) of the biosensor-expressing motor neurons (MNs) measured at different time points of the differentiation protocol. Values were obtained by analyzing the microscopic images of the respective MNs. (**A**) Day 29 of differentiation. (**B**) Day 20 of differentiation. (**C**) Day 29 of differentiation. SOD1-G128R MNs were cultured with or without neurotrophic factors (NTFs) during maturation. (**D**) Day 29 of differentiation. The standard neuronal medium was replaced by the nutrient-deprived (-B-27) medium 24 h before the measurement. Data (N = 10–15, and 18 for B-27 deprived samples, fields of view, collected from MNs, derived from three iPSC lines with the same genotype) are the mean ± standard deviation *p < 0.05, **p < 0.01, ****p < 0.0001, one-way ANOVA with post hoc Dunnet’s tests for (**A**), (**B**), and (**D**), Welch t-test for (**C**). The same datasets of the cytoplasmic and mitochondrial H_2_O_2_ levels in SOD1-G128R (-NTF) were used in (**A**) and (**C**).

The basal level of H_2_O_2_ reflects stationary redox balance and the general condition of the cell. The H_2_O_2_ biosensor allows us to estimate the relative amount of H_2_O_2_ molecules in the compartment and determine if it is different from the control. We performed live imaging of the mature MNs (Day 29) at the end of the differentiation protocol to obtain information about their redox state. We did not observe any signs of pathological oxidation in the cytoplasm and mitochondria of the SOD1-D91A and iALS MNs. SOD1-G128R MNs, however, demonstrated a 2.7 times higher level of the cytoplasmic and five times higher level of the mitochondrial H_2_O_2_ compared to the control (Fig. 4A). Notably, the cytoplasmic oxidation measured at the stage of immature MNs (Day 20) was similar for all neurons, suggesting that the SOD1-G128R MNs hyper oxidation develops with the maturation of the MNs (Fig. 4B). To correct the observed phenotype, we added a combination of neurotrophic factors (NTFs, See Materials and Methods) to the culture medium during SOD1-G128R MNs maturation (differentiation days 19–29). This resulted in a significant decrease in cytoplasmic H_2_O_2_ to the normal level. However, it did not affect the mitochondrial level of H_2_O_2_ (Fig. 4C).

To put additional stress on the MNs, we depleted culturing medium from the majority of the nutrients by removing the B-27 supplement (chemically-defined mixture of antioxidant enzymes, proteins, vitamins, and fatty acids) 24 h before live imaging and measured the cytoplasmic and mitochondrial H_2_O_2_ levels. We discovered that B-27 deprivation did not influence the cytoplasmic level of H_2_O_2_ (Fig. 4A,D, Supplementary Fig. S9). However, MNs cultured in the absence of B-27 demonstrated a significant increase in the mitochondrial H_2_O_2_ level, which was, on average, four times higher than in the cytoplasm of the corresponding neurons. For the SOD1-G128R MNs, the B-27 deprivation resulted in an additional increase in both cytoplasmic and mitochondrial H_2_O_2_ levels (Fig. 4D, Supplementary Fig. S9).

Interestingly, the H_2_O_2_ levels in the cytoplasm and mitochondria of mature iALS MNs were lower compared to the control, probably due to the different origin of the iALS cell line (Fig. 4A,D).

### Antioxidants removal from the medium induces H_2_O_2_ accumulation in the cytoplasm of motor neurons

**Figure 5:**
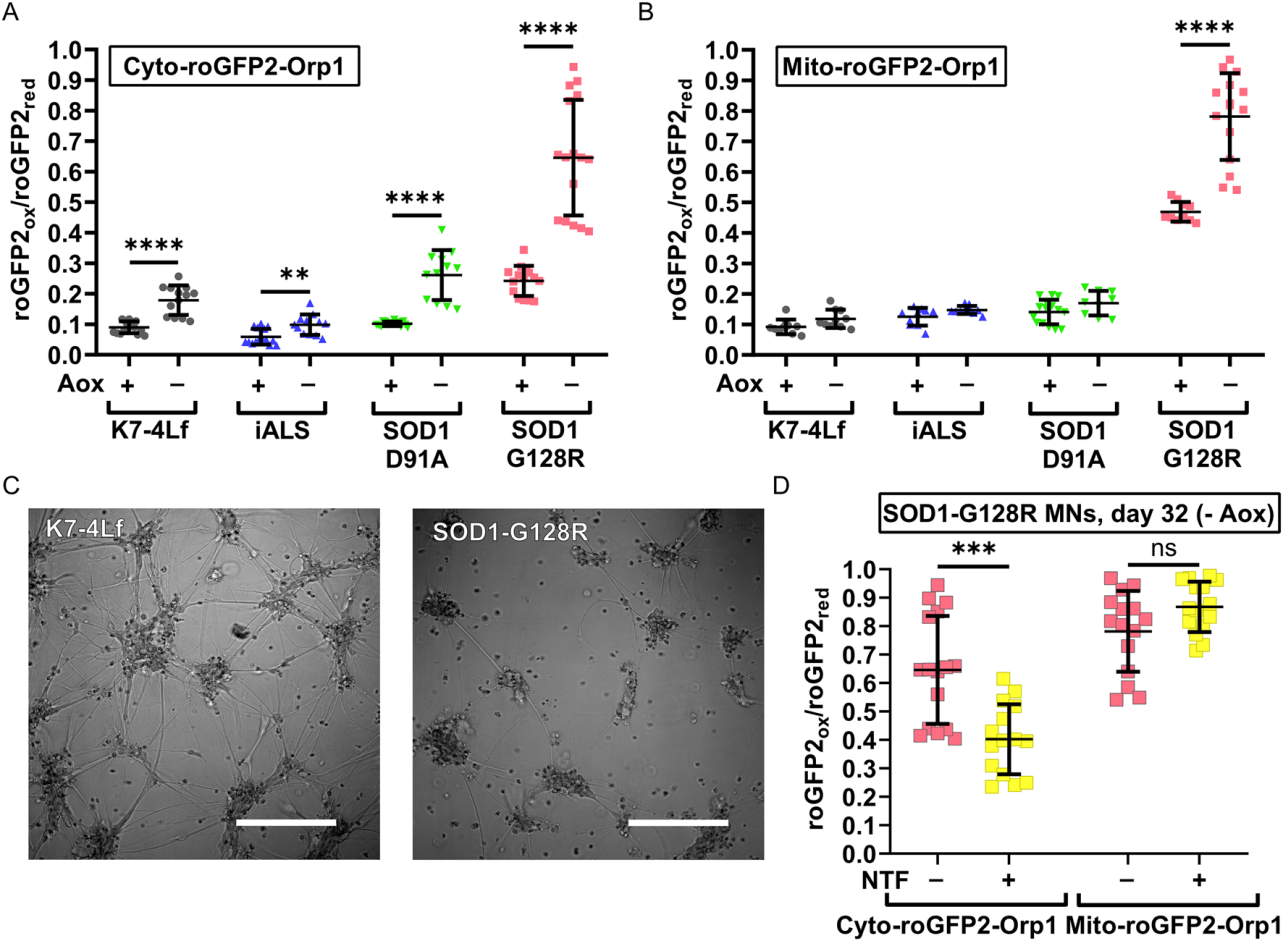
Effect of the antioxidant deprivation on the basal H_2_O_2_ level in the cytoplasm and mitochondria of motor neurons (MNs). Values were obtained by analyzing the microscopic images of the respective MNs. Basal levels of H_2_O_2_ in the cytoplasm (**A**) and mitochondria (**B**) of MNs were measured on the differentiation days 29 (Aox+) and 32 (Aox−). C Representative images of the K7-4Lf and SOD1-G128R MN morphology in the antioxidant-free medium (differentiation day 32, DIC contrast), scale bar 200 μm. (**D**) Basal levels of H_2_O_2_, measured on the differentiation day 32 in SOD1-G128R MNs cultured with or without neurotrophic factors (NTFs) in the culture medium during maturation. Data (N = 10–16 fields of view, collected from MNs, derived from three iPSC lines with the same genotype) are the mean ± standard deviation. Ns—non significant, **p < 0.01,***p < 0.001, ****p < 0.0001, Welch t-test. The same datasets of cytoplasmic and mitochondrial H_2_O_2_ levels in SOD1-G128R (-NTF) were used in (**A**–**C**). The datasets used to present the cytoplasmic and mitochondrial oxidation on the differentiation day 29 (Aox+) for K7-4Lf, iALS, SOD1-D91A, and SOD1-G128R MNs in the panels A and B are the same as in Fig. 4A.

Amongst many additives, the neuronal medium contains antioxidants, such as ascorbic acid, vitamin E, vitamin E acetate, superoxide dismutase, catalase, and glutathione, acting against ROS that appear in the medium during in vitro culturing. The removal of the antioxidants from the medium may force MNs to rely on endogenous antioxidant systems for ROS neutralization, making the mutant MNs more vulnerable. We cultured the neurons in the antioxidant-free medium (without ascorbic acid, and using the B-27 supplement without antioxidants) for three days in addition to the differentiation protocol and performed live imaging of the MNs (Day 32, Fig. 5A,B). All MNs showed an increase in the cytoplasmic H_2_O_2_ level by 1.5–2.5 times. However, only in the SOD1-G128R MNs the mitochondrial level of H_2_O_2_ increased along with the cytoplasmic level. In addition, SOD1-G128R MNs also demonstrated visible changes in the morphology with the axon attrition and cytoplasmic vacuolization (Fig. 5C). Culturing of SOD1-G128R MNs with the NTFs reduced oxidation of the cytoplasm, but it failed to keep it to the normal level. Moreover, the mitochondrial level of H_2_O_2_ was unaffected by addition of the NTFs (Fig. 5D).

### Cyto-roGFP2-Orp1 biosensor, expressed in motor neurons, reflects the kinetics of H_2_O_2_ neutralization

**Figure 6:**
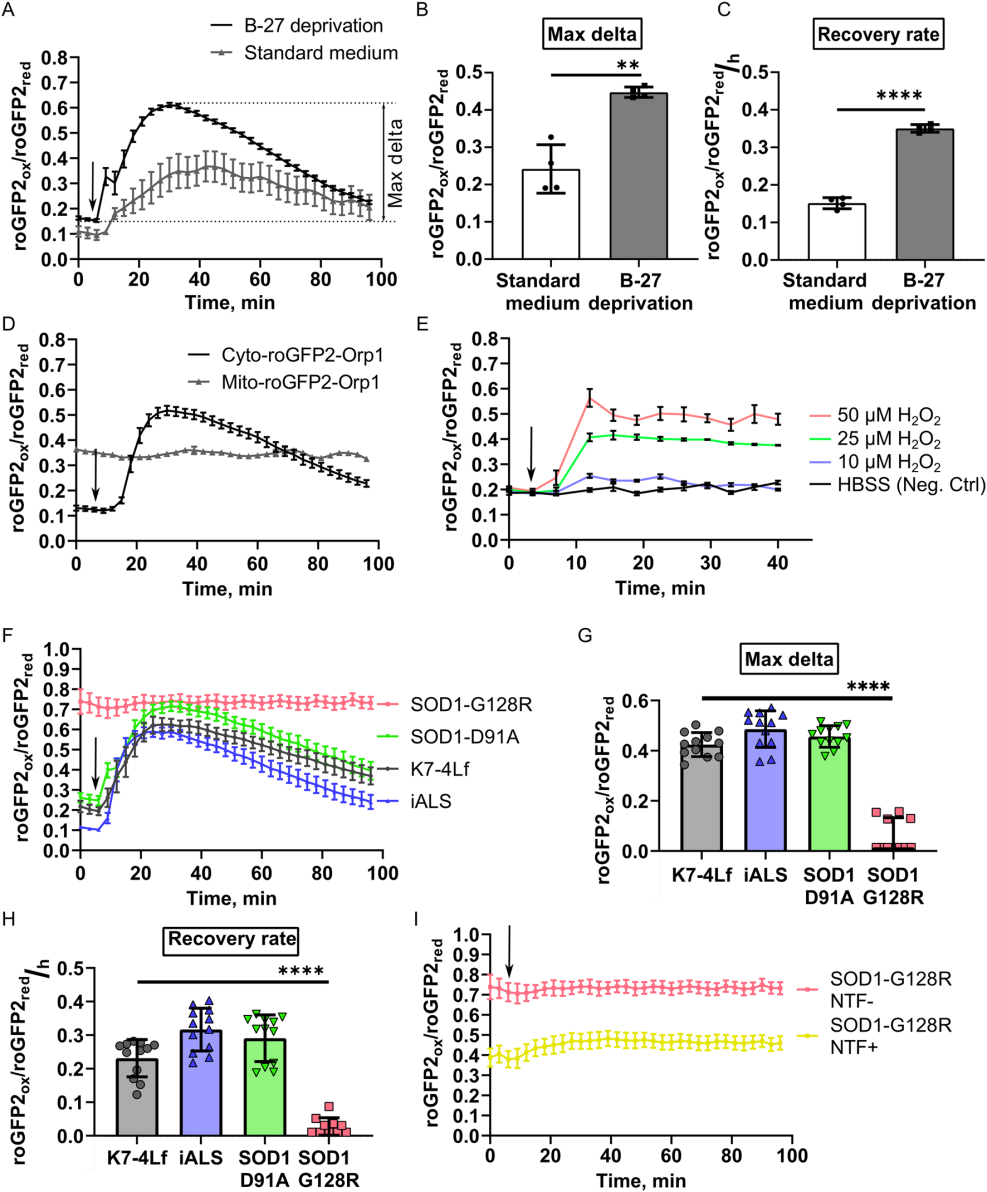
Kinetics of H_2_O_2_ utilization in live iPSC-derived motor neurons (MNs). Values were obtained by analyzing the microscopic images of the respective MNs. (**A**) Response of the MNs, cultured in the standard or nutrient-deprived (without B-27) medium to addition of 10 μM H_2_O_2_. Data (N = 4 fields of view) are the mean ± standard error of the mean (S.E.M.). Max delta (difference between the maximum and initial level of oxidation, defined in panel A) of the cytoplasm oxidation (**B**) and recovery rate (**C**) of the MNs, expressing Cyto-roGFP2-Orp1, after H_2_O_2_ addition. Data (N = 4 fields of view) are the mean ± standard deviation (S.D.) (**D**) Response of the MNs, expressing Cyto-roGFP2-Orp1 or Mito-roGFP2-Orp1, to addition of 10 μM H_2_O_2_. Data (N = 4 fields of view) are the mean ± S.D. (**E**) Response of the MNs, expressing Mito-roGFP2-Orp1, to the different H_2_O_2_ concentrations. Data (N = 3 fields of view) are the mean ± S.D. (**F**) Response of the K7-4Lf, iALS, SOD1-D91A, and SOD1-G128R MNs, expressing Cyto-roGFP2-Orp1 to addition of 10 μM H_2_O_2_. Data (N = 12 fields of view, collected from MNs, derived from three iPSC lines with the same genotype) are the mean ± S.E.M. Max delta of the cytoplasm oxidation (**G**) and recovery rate (**H**) of the K7-4Lf, iALS, SOD1-D91A, and SOD1-G128R MNs, expressing Cyto-roGFP2-Orp1 after 10 μM H_2_O_2_ addition. Negative values in the SOD1-G128R MNs were set to 0 on the graphs. Data (N = 12 fields of view, collected from MNs, derived from three iPSC lines with the same genotype) are the mean ± S.D. (**I**) Response of the SOD1-G128R MNs, expressing Cyto-roGFP2-Orp1 cultured in the standard medium (NTF−) or medium supplemented with neurotrophic factors (NTF+) to 10 μM H_2_O_2_. Data (N = 4 fields of view) are the mean ± S.E.M. The moment of the H_2_O_2_ addition is marked by an arrow. **p < 0.01, ****p < 0.0001. Welch t-test for (**B**), and (**C**), one-way ANOVA with post hoc Dunnet’s tests for (**G**) and (**H**). The same dataset was used in Figs. 7F,I to represent the SOD1-G128R (− NTF) reaction to H_2_O_2_.

To assess the dynamic response of the MNs expressing Cyto-roGFP2-Orp1 and Mito-roGFP2-Orp1 biosensors to oxidation, we first determined the concentration of H_2_O_2_ that did not affect MN viability in the culture. Only treatment with 10 µM H_2_O_2_ did not significantly affect the viability of the tested MNs (Supplementary Fig. S10), which was consistent with the previously published data^40,41^. Next, we tested whether culturing in the standard neuronal medium distorts the cellular response to the H_2_O_2_ and performed a live recording of the Cyto-roGFP2-Orp1-expressing MNs reaction to the addition of 10 μM H_2_O_2_. These MNs were either cultured in the standard differentiation medium or starved in the nutrient-deprived medium before the measurement (Fig. 6A). The overall reaction of the cells was similar: MNs expressing Cyto-roGFP2-Orp1 displayed oxidation followed by slow reduction, reflecting the change in the cytoplasmic H_2_O_2_ level. However, the reaction of MNs cultured in the nutrient-deprived medium before the experiment was more prominent. We detected a higher delta of the biosensor oxidation and faster reduction compared to the non-starved MNs (Fig. 6B,C). Since components present in the standard medium affected the cellular reaction, we conducted further measurements of the dynamic response on the cells that were starved before the experiment. Using the parameters established earlier, we recorded the reaction of MNs expressing the Mito-roGFP2-Orp1 biosensor to 10 μM H_2_O_2_ in real-time. We did not detect any response of the Mito-roGFP2-Orp1 biosensor to the exogenous H_2_O_2_. An oxidation value of the biosensor remained constant during imaging, suggesting that the mitochondrial H_2_O_2_ level was also stable (Fig. 6D). The addition of H_2_O_2_ in higher concentrations (25 μM and 50 μM) induced mitochondrial oxidation but damaged the neurons. With this finding, we decided not to measure the dynamic response for the Mito-roGFP2-Orp1 sensor (Fig. 6E).

Next, we analyzed how the mutations introduced in *SOD1* affected neuronal reaction to exogenous H_2_O_2_. We did not find any differences in H_2_O_2_ utilization in the cytoplasm of Cyto-roGFP2-Orp1-expressing SOD1-D91A, iALS, and control (K7-4Lf) MNs (Fig. 6F–H). Analysis of the SOD1-G128R MNs response to the H_2_O_2_ revealed an aberrant reaction. Due to the high initial oxidation of the MNs, cells did not respond to the exogenous H_2_O_2_ (Fig. 6F–H). Although culturing of the SOD1-G128R MNs with the NTFs slightly reduced initial oxidation of the cytoplasm, it did not affect the cellular reaction to the H_2_O_2._ The Cyto-roGFP2-Orp1-expressing MNs demonstrated moderate oxidation of the cytoplasm without signs of subsequent reduction (Fig. 6I).

### Motor neurons expressing the Cyto-roGFP2-Orp1 biosensor accumulate H_2_O_2_ in the cytoplasm due to glutamate-induced excitotoxicity

**Figure 7:**
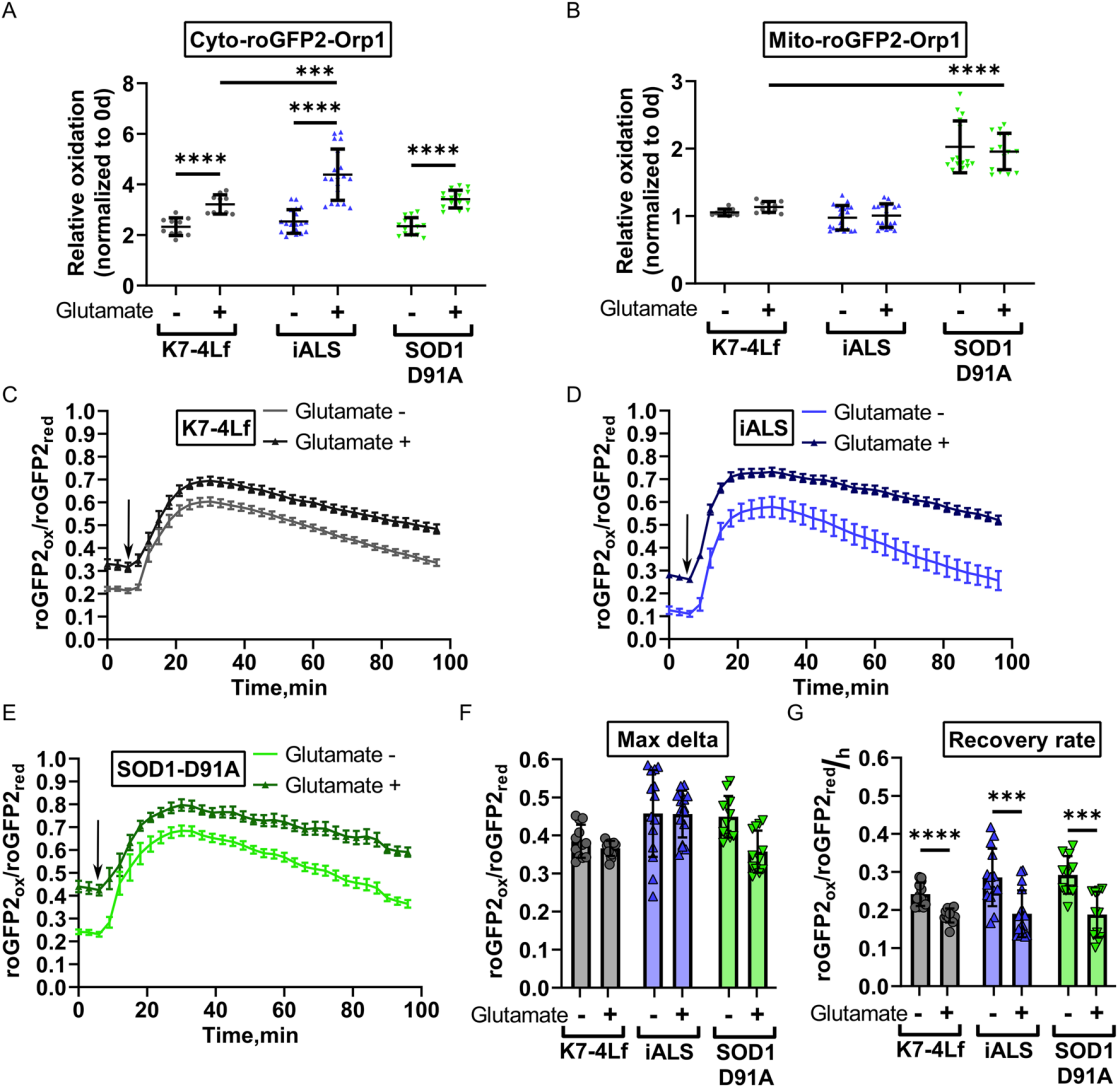
The reaction of iPSC-derived motor neurons (MNs) to excitotoxicity. Values were obtained by analyzing the microscopic images of the respective MNs. Basal level of H_2_O_2_ in the cytoplasm (**A**) and mitochondria (**B**) of MNs after 5-days incubation with monosodium glutamate (20 μM) + PDC (100 μM). Data (N = 10–15 fields of view, collected from MNs, derived from three iPSC lines with the same genotype) are normalized to the basal H_2_O_2_ level measured before the addition of glutamate and presented as the mean ± standard deviation (S.D.). The response of K7-4Lf (**C**), iALS (**D**), and SOD1-D91A (**E**) MNs to addition of 10 μM H_2_O_2_ (marked by an arrow). The reaction of MNs cultured with glutamate is depicted in the darker colors. Data (N = 12 fields of view, collected from MNs, derived from three iPSC lines with the same genotype) are the mean ± standard error of the mean (S.E.M.) Max delta (difference between maximum and initial level of oxidation) of the cytoplasm oxidation (**F**) and recovery rate (**G**) of the MNs expressing Cyto-roGFP2-Orp1 after addition of 10 μM H_2_O_2_. Data (N = 12 fields of view, collected from MNs, derived from three iPSC lines with the same genotype) are the mean ± S.D. *p < 0.05, ***p < 0.001, ****p < 0.0001, Welch t-test for paired comparisons in (**A**, **B**, **F**, **G**), one-way ANOVA with post hoc Dunnet’s tests for comparisons between MNs, treated with glutamate in (**A**) and (**B**).

Glutamate excitotoxicity is a known pathological hallmark of neurodegeneration^42^. To test whether the Cyto-roGFP2-Orp1 and Mito-roGFP2-Orp1 biosensors can reflect redox imbalance caused by the excitotoxicity, we incubated MNs, expressing these biosensors with monosodium glutamate (20 μM) and the glutamate reuptake inhibitor (PDC, 100 μM) for five days and measured the cytoplasmic and mitochondrial H_2_O_2_ levels. Since SOD1-G128R MNs died shortly after the beginning of the experiment due to reduced viability, the measurement was conducted only for K7-4Lf, SOD1-D91A, and iALS MNs. We discovered that the glutamate treatment induced the accumulation of H_2_O_2_ in the cytoplasm regardless of the MN genotype (Fig. 7A). Cytoplasmic oxidation in iALS MNs treated with glutamate was higher compared to the glutamate-treated control MNs. However, we did not find the same for the SOD1-D91A MNs (Fig. 7A), indicating that this effect was not due to the *SOD1* mutation. We did not observe the same for the mitochondria, despite the known connection of mitochondrial dysfunction with excitotoxicity (Fig. 7A)^43^. The mitochondrial oxidation in SOD1-D91A MNs in both control and glutamate treated samples was increased (Fig. 7B), although we were unable to determine if the oxidation was a hallmark of the *SOD1* mutation or a technical artifact.

Further, we investigated how incubation with monosodium glutamate affected the dynamics of H_2_O_2_ utilization in the cytoplasm of the Cyto-roGFP2-Orp1-expressing MNs. We found that MNs treated with glutamate had a reduced recovery rate after the H_2_O_2_ addition compared to the non-treated sample (Fig. 7C–G).

## Discussion

Nowadays, genetically encoded biosensors are frequently applied to research physiological and pathological processes^44,45^, replacing molecular probes. Indeed, they have provided extensive information about the dynamics of redox balance components, e.g., H_2_O_2_ or GSH/GSSG ratio, in different cell compartments and tissues^22,46^. Since the biosensor molecules are delivered inside the cells as plasmids for temporal expression or as viral vectors for sequence integration in the genome, the robustness of biosensors-related studies often relies on the efficiency of delivery and location of transgene integration. Here, we tried to improve the existing approach for biosensor experiments by targeted insertion of the cytoplasmic and mitochondrial H_2_O_2_ biosensors in combination with their controlled expression (Fig. 1A). This approach allowed us to obtain iPSC lines, stably expressing the biosensors, and avoid potential undesirable effects of random integration (Fig. 2). For the first time, to our knowledge, we showed that the biosensor expressing in such a system is a reliable method to study MNs in vitro, as the intensity of the signal generated from a single copy of the biosensor and the dynamic range of that signal, are sufficient for routine imaging (Supplementary Fig. S8). However, we found that the *AAVS1* locus does not support the stable expression of the integrated biosensors in MNs despite its “safe harbor” status, probably, due to chromatin remodeling occurring during differentiation^47^. Although constant activation of the biosensor promoter during differentiation, applied here, prevented it from silencing, this in itself emphasizes the necessity to discover new safe harbors in the human genome (Fig. 3C–E).

The measurements, conducted in MNs with Cyto-roGFP2-Orp1 and Mito-roGFP2-Orp1 biosensors, revealed that the basal level of cytoplasmic and mitochondrial oxidation is dynamic and serves as an indicator of cellular distress. Nutrient deprivation, known to induce moderate stress, activates autophagy through the ROS signaling^48–50^. In our data, MNs deprived of nutrients demonstrated additional oxidation of the mitochondria (Fig. 4), which was in line with the previously published data^48^. Additionally, a stable cytoplasmic level of H_2_O_2_ in this condition also suggests that this is a part of a natural response. Interestingly, despite the stability of the cytoplasmic H_2_O_2_ level in the nutrient-deprived MNs, the reaction of the cells to exogenous H_2_O_2_ was more prominent. Specifically, a higher amplitude of oxidation and faster recovery were recorded (Fig. 6A–C), implying that in these moderate stress conditions, the buffering capacity of the cytoplasm is lower, but the antioxidant system is in the mobilized, more active state^51^.

Differences in cytoplasmic and mitochondrial reactions to the exogenous H_2_O_2_ reflect relative independence of the mitochondrial and cytoplasmic antioxidant systems (Fig. 6D–E). The absence of significant changes in the signal of the Mito-roGFP2-Orp1 sensor and the presence of a normal reaction of the Cyto-roGFP2-Orp1 sensor in response to 10 μM H_2_O_2_ suggests that the cytoplasmic antioxidant system neutralizes most exogenous H_2_O_2_ molecules. The reaction that only appears in response to a lethal amount of H_2_O_2_ suggests that exceeding hydrogen peroxide level in the cytoplasm above the value that it can quickly neutralize leads to a transfer of H_2_O_2_ molecules into mitochondria and apoptosis induction^52^.

To test if the developed platform can reflect pathological oxidation in MNs caused by a genetic mutation, we generated isogenic cell lines with *SOD1* mutations of, presumably, different severity: D91A and G128R^31,32^. Since no homozygous variants have been found, we selected the lines that have one allele with the target mutation and the other with large deletion (SOD1-D91A) or premature termination codon (SOD1-G128R) to maximize potential damage and make the pathological phenotype more perceptible (Fig. 1B,C). While the neurons generated from iPSC lines with a D91A mutation were not different from the control, MNs derived from the SOD1-G128R iPSCs demonstrated higher levels of oxidation in both the cytoplasm and mitochondria, which only increased in stress conditions, such as nutrient or antioxidant deprivation (Figs. 4, 5, 6). Through live imaging of the neurons in the different stages of differentiation, we were able to detect that described pathological features only appear in the latest stages of differentiation (Fig. 4B), which is consistent with the fact that ALS does not manifest until a certain age^53^. Using the separate measurement of H_2_O_2_ in different compartments, we were able to detect that mitochondrial oxidation in mature SOD1-G128R MNs was more pronounced than that of the cytoplasm (Figs. 4A, 5A,B), suggesting that accumulation of H_2_O_2_ in mitochondria precedes its accumulation in the cytoplasm. Together with the slower axon growth (Fig. 3B), this could be a sign of mitochondrial dysfunction that has been linked to neurodegeneration before^1,54^. Moreover, the inability of neurotrophic factors to correct this phenotype indicates the severity and irreversibility of the cellular condition observed at this point (Figs. 4C, 5D).

Glutamate excitotoxicity is one of the major mechanisms of ALS development, in which excessive activation of the glutamate receptors leads to mitochondrial dysfunction and apoptosis^42,52^. It is known that this process is accompanied by increased ROS production, which connects it with oxidative stress^55^. In our study, we, for the first time, observed in live MNs how glutamate-induced excitotoxicity affected oxidation in the neuronal compartments. Glutamate-treated neurons demonstrated accumulation of H_2_O_2_ in the cytoplasm and a slower recovery rate after H_2_O_2_ addition (Fig. 7). However, no differences between the D91A mutant and control MNs were observed, suggesting that the power of stress applied or the degree of MN maturation was insufficient for the pathological phenotype to manifest, meaning that further optimization of the study parameters is required.

In conclusion, our work presents a new approach for the application of cell-based disease models for research that can be used to generate other similar models. We expect this approach to be expanded to include other disease-associated mutations or the biosensors of other cellular processes. There are, however, still elements that require further optimization, such as research for the new safe-harbor loci that sustain transgene expression. Moreover, the culture conditions applied for the study must be considered since modern cell culture systems are highly protective and can hinder the results of the measurements. We also look forward to the application of automated cell imaging, allowing continuous measurement during cell differentiation and maturation, which could also be beneficial for more complex research and screening for future therapeutics.

## Methods

### IPS cell culture

iPSCs were maintained on the layer of mitotically inactivated mouse embryonic fibroblasts in KnockOut DMEM (Gibco) with 15% knockout serum replacement (Gibco), 0.1 mM non-essential amino acids (Gibco), penicillin/streptomycin (Lonza), 1 mM GlutaMAX-I, and 10 ng/mL bFGF at 37 °C and 5% CO_2_. IPSCs were dissociated with TrypLE (Gibco) and split at 1:10 twice a week in the iPSC medium supplemented with 10 ng/ml Y-27632. Original iPSC lines were derived from the patient (iALS) with a diagnosed hereditary form of ALS^37^ and a healthy individual (K7-4Lf) who had no associations with any genetic disease^33^ (Supplementary Table S2). The use of the iPSC lines, generated from the patients’ materials, in the study has been approved by the Research Ethics Committee of FSBI Federal Neurosurgical Center (Novosibirsk, Russia), protocol number 1 14/03/2017.

### Generation of iPSC lines with the c.272A>C (D91A) and c.382G>C (G128R) substitutions in *SOD1*

The guide RNAs (gRNAs) targeting sequences in exons 4 and 5 of the *SOD1* gene and the *AAVS1* locus were designed using the web-based tool http://crispr.mit.edu^56^. The Alt-R^®^ crisprRNA and tracrRNA were obtained from IDT (Integrated DNA technologies), and Cas9 protein was expressed in *E. coli* and purified according to the previously published protocol^57^. The CRISPR/Cas9 ribonucleoprotein (RNP) complexes were assembled according to the manufacturer’s instructions on the day of transfection. For the introduction of the c.272A>C and c.382G>C mutations we used appropriate RNP (20 pmol tracrRNA + 20 pmol crisprRNA (SOD1-4/SOD1-5) + 20 pmol Cas9) complexes mixed with 100 pmol of D91A ssODN (single-stranded oligodeoxynucleotide) or G128R ssODN donor. The cells were passed 24 h before the transfection in the iPSC medium supplemented with Y-27632 (10 ng/ml). Transfection was performed using Neon Transfection System 10 μl Kit (Invitrogen) according to the manufacturer’s instructions. The cells were seeded on feeder-coated 4 cm^2^ dishes in the iPSC medium supplemented with Y-27632 (10 ng/ml). The next day, the cells were dissociated with TrypLE, strained through the cell strainer, and subcloned on 96-well plates for propagation and analysis. Genomic DNA of the survived clones was obtained and analyzed for the presence of the target mutations.

To detect the c.272A>C mutation, we designed primers for tetra-primer ARMS (amplification-refractory mutation system) PCR screening using http://primer1.soton.ac.uk/primer1.html^58^ and performed touchdown 3-step PCR with annealing at 68–64 °C for 9 cycles, then at 64 °C for 21 cycles. The PCR products were analyzed in 2% agarose gel. Clones positive for the mutant allele were further examined by Sanger sequencing. To detect the c.382G>C mutation, we designed a pair of primers that amplify the target locus of the *SOD1* gene and two fluorescent probes targeting either wild-type or mutant sequences. Using LightCycler 480 (Roche), we analyzed the clones and selected those who had strong signals from the mutant-targeted probe. The target mutation was further confirmed by Sanger sequencing. Potential off-target CRISPR/Cas9 sites were determined using the Benchling algorithm (https://www.benchling.com/), and the top 5 hits were then investigated in the obtained cell lines by Sanger sequencing. Clones used in the experiments were characterized according to the Human Pluripotent Stem Cell Registry standards with the protocols described previously^34^. All oligonucleotides/primers used in the study are listed in the Supplementary Table S1.

### Generation of iPSC lines with target *AAVS1* inserts

To insert Cyto-roGFP2-Orp1, Mito-roGFP2-Orp1 and transactivator in *AAVS1*, we used AAVS1 RNP (100 pmol tracrRNA + 100 pmol AAVS1 crRNA + 100 pmol Cas9) mixed with 5 μg of donor plasmids mix, containing equimolar amounts of transactivator donor (pAAVS1-Neo-M2rtTA, Addgene # 60843) + pCyto-roGFP2-Orp1-donor or pMito-roGFP2-Orp1-donor. The transfection was performed using Neon Transfection System 100 μl Kit according to instructions. The cells were then seeded on feeder-coated 10 cm^2^ dishes in the iPSC medium supplemented with Y-27632 (10 ng/ml) and maintained before the selection until small colonies formed. We supplemented the iPSC medium with puromycin dihydrochloride for 3 days to select subclones with the target inserts. Then we replaced the antibiotic with neomycin sulfate and incubated the cells for 4–5 more days. Antibiotic concentrations were determined for each cell line before the experiment. At the end of the selection, we added doxycycline hyclate (2 μg/ml) and examined the remained clones for the presence of fluorescent signal from the biosensors’ roGFP2 using the Nikon Eclipse Ti2-E microscope. The clones positive for roGFP2 expression that survived double antibiotic selection were manually harvested into separate dishes for maintenance and analysis. Genomic DNA extracted from these iPSC clones was analyzed for the presence of the target and off-target inserts of the donor plasmids using PCR (Supplementary Table S1).

### Co-localization of the roGFP2 fluorescent signal with the cytoplasm and mitochondria

The cells were seeded on Matrigel-coated cell imaging coverglasses in the media, supplemented with doxycycline hyclate (2 μg/ml). On the day of imaging, the cells were incubated in HBSS + Ca^2+^, Mg^2+^, supplemented with cell-permeant nuclear counterstain (NucBlue™ Live ReadyProbes™ Reagent, 2 drops/ml, Thermo Fisher Scientific) and 250 nM tetramethylrhodamine, methyl ester, perchlorate (TMRM, Thermo Fisher Scientific) for mitochondrial staining, or 1 μM CellTracker™ Red CMTPX Dye (Thermo Fisher Scientific) for cytoplasmic staining, for 30 min. Then, cells were washed with HBSS + Ca^2+^, Mg^2+^ and immediately visualized. Micrographs were captured using the LSM-780 (Zeiss) microscope and ZEN black software.

### Immunocytochemistry

The cells were fixed in 4% formaldehyde solution for 10 min at room temperature (RT), permeabilized with 0.5% Triton X-100 for 30 min at RT, and then incubated with blocking buffer (1% bovine serum albumin (BSA) in PBS) for 30 min at RT. After, the cells were incubated with specific primary antibodies overnight at 4 °C. Next, the appropriate secondary antibodies were added for 1.5–2 h incubation at RT. All antibodies were diluted in blocking buffer, and the cell nuclei were visualized with DAPI (1 μg/ml solution in PBS). The antibodies and their dilution ratios are listed in Supplementary Table S11. Micrographs were captured using either Nikon eclipse Ti-E microscope (Nikon) and NIS Elements software or the LSM-780 (Zeiss) microscope and ZEN black software.

### Motor neuron differentiation and maintenance

The iPSCs were seeded on Matrigel-coated dishes in E8 (Gibco) medium and maintained in feeder-free conditions for at least 2 passages prior to differentiation. MN differentiation was performed according to the previously published protocol^38^. For neural patterning, the E8 medium was changed to neuronal differentiation medium (NDM): F12/DMEM:Neurobasal—50:50, 0.5 × N2 supplement, 0.5 × B-27 supplement, 2 mM GlutaMAX, and 0.1 mM ascorbic acid. For the first six days NDM was supplemented with 2 μM CHIR99021 (StemRD), 2 μM SB431242 (Selleckchem), and 2 μM DMH1 (Tocris). Following this, neuroepithelial cells were dissociated 1:6 with Accutase (Gibco) and maintained for six days in NDM supplemented with CHIR99021, SB431242, DMH1, 0.5 μM Purmorphamine (Stemgent), and 0.1 μM retinoic acid (Sigma). Then, the MN progenitors were dissociated with Accutase and cultured in suspension on agarose-coated (non-adherent) dishes in NDM, supplemented with 0.1 μM Puromorphamin and 0.5 μM retinoic acid for 6 days. For maturation, cells were dissociated with Accutase to a single-cell suspension, strained through the 70 μm cell strainer, and seeded in NDM, supplemented with 0.1 μM Puromorphamin, 0.5 μM retinoic acid, and 0.15 μM Compound E (EMDMillipore). Depending on the experiment, MNs were seeded in different settings: for flow cytometry and mRNA expression analysis, we seeded 1.5 × 10^5^ cells/cm^2^ on Matrigel-coated 60 mm Petri dishes; for immunocytochemistry—5 × 10^4^ cells/cm^2^—on Matrigel-coated cell imaging coverglasses (Eppendorf); for axon measurement—1.5 × 10^4^ cells/cm^2^—on Matrigel-coated cell imaging coverglasses; for biosensors live imaging—inside Matrigel layer (see “Supplementary methods”, Supplementary Fig. S12). After each dissociation, cells were transferred to Y-27632-supplemented (10 ng/ml) medium, which was replaced daily. The cells were also supplemented with doxycycline (2 μg/ml) every other day during the differentiation unless otherwise indicated.

For starvation (B-27 deprivation) induction, standard NDM was replaced with nutrient-deprived medium (F12/DMEM:Neurobasal—50:50, 1 × N2 Supplement, 2 μg/ml doxycycline hyclate). For the antioxidant-deprivation assay and excitotoxicity induction assay, MNs were incubated in antioxidant-free medium (F/D:Neurobasal—50:50, 0.5 × N2 supplement, 0.5 × B-27 supplement without antioxidants, 2 μg/ml doxycycline hyclate, 0.5 μM retinoic acid, and 0.15 μM Compound E). For studying the neurotrophic factor effect in MN oxidation, cells were cultured in the respective medium supplemented with IGF1 (PeproTech, 10 ng/ml), CNTF (PeproTech, 10 ng/ml), and BDNF (PeproTech, 10 ng/ml).

### Reverse-transcription quantitative PCR (RT-qPCR)

1 μg of total RNA extracted with Trizol reagent (Invitrogen) was used for reverse transcription with M-MuLV-RH reverse transcriptase (Biolabmix) and random hexamer primer (Invitrogen) and diluted 1:10 in MilliQ H_2_O. qPCR analysis was performed using the LightCycler 480 (Roche). Gene expression of iPSC markers in the *SOD1* mutant iPSCs was normalized to the *B2M* housekeeping gene and compared to the original iPSC line using the ΔΔCt-method. *ISL1*, *CHAT,* and *MNX1* genes expression in MNs was normalized to the mean of the *GAPDH*, *HPRT1,* and *RPL13* housekeeping genes and compared to that of the iPSC using the ΔΔCt-method. The roGFP2 and rtTA transgenes expression in MNs and iPSCs was normalized to the mean of *GAPDH*, *HPRT1*, and *RPL13* housekeeping genes and compared to the expression in MNs that were not treated with doxycycline during differentiation. The primers used are listed in Supplementary Table S2.

### Flow cytometry analysis

The cells were dissociated with Accutase on day 20 of the differentiation protocol, resuspended in cold PBS, and centrifuged at 400*g* for 5 min (the same settings were used for all subsequent centrifugation steps). The pellet was resuspended in 1 ml cold 4% formaldehyde solution and incubated on ice for 10–15 min. Next, we added 1 ml cold PBS, centrifuged the cells, discarded supernatant, resuspended the pellet in 1 ml ice-cold 100% methanol, and incubated it for 10–15 min on ice. Then, the pellet was washed twice with flow cytometry staining buffer (1% BSA, 0.2 μM EDTA, in PBS) and resuspended in it to 1 × 10^6^ cells/ml concentration. 100 μl of the cell suspension (1 × 10^6^ cells/ml) was incubated with anti-ISL primary antibodies overnight at 4 °C. The cells were washed with flow cytometry staining buffer, incubated with the secondary antibodies for 30 min at RT, and analyzed using FACSAria (BD Biosciences). Unlabeled cells and isotype-labeled cells were used as controls.

### Fluorescence intensity measurement

MN images (four for each sample) were obtained using the Zeiss LSM-780 confocal laser scanning microscope (Pan-Apochromat 20 × objective) adjusted for visualization of a green dye (excitation—488 nm; emission collection at 500–530 nm). Mean intensity of the fluorescence on each image was measured using the ImageJ software and corrected total cell fluorescence (CTCF) was calculated with the formula: CTCF = Integrated Density − (Area occupied by cells × Mean fluorescence of background readings).

### Axon measurement

Immature MNs were seeded on the cell imaging coverglasses and grown for two days in a medium supplemented with a neurotrophic factor (NTF) cocktail: IGF1 (PeproTech, 10 ng/ml), CNTF (PeproTech, 10 ng/ml), and BDNF (PeproTech, 10 ng/ml). Doxycycline (2 μg/ml) was added to induce the roGFP2 expression. Images were obtained with the Nikon Eclipse Ti-2E microscope (20 × objective, FITC channel). Using ImageJ, we manually measured the length of the longest processes (axons) of free-lying neurons with visible ends. Only axons with a length more than twice the size of the neurons’ bodies were considered for the analysis. If the neuron had two long processes, the longest one was considered for the measurement. The mean length of the axons was calculated based on the data obtained from the differentiation of three separate iPSC clones for each genotype.

### Image acquisition

The general procedures for the image acquisition and data analysis, including samples and solution preparations, microscopy settings, biosensors’ calibration, basal H_2_O_2_ measurement, H_2_O_2_ utilization analysis, and data normalization, are described in the “Supplementary methods”.

### XTT viability assay

The viability of the MNs was assessed 3 h after the addition of H_2_O_2_ in different concentrations (10 μM, 25 μM, 50 μM, and 100 μM) by an XTT test with 2,3-bis-(2-methoxy-4-nitro-5-sulfophenyl)-2H-tetrazolium-5-carboxanilide (Roche), according to the manufacturer’s instructions at 20 h after the reagents were added. Cells were seeded on Matrigel-covered 96-well plates (1.6 × 10^4^ cells/well). Before adding H_2_O_2_, we replaced the neuronal medium with HBSS + Ca^2+^ + Mg^2+^. For each H_2_O_2_ concentration, the experiments were carried out in three replicates. The viability data were normalized to the values obtained in the control well, treated with PBS and analyzed by Welch t-test.

### Excitotoxicity induction assay

MNs were incubated in the neuronal maintenance medium supplemented with 20 μM monosodium glutamate (Sigma-Aldrich) and 100 μM l-trans-pyrrolidine-2,4-dicarboxylic acid (PDC, Sigma-Aldrich) for 5 days, changing the medium every other day. After 5 days of incubation, we obtained images of the treated MNs and non-treated control MNs. The data obtained at the end of the experiment were normalized to the starting oxidation values, measured before the glutamate addition, to describe the changes that emerged during the experiment.

### Statistics

Graphs and statistical analyses were performed in GraphPad Prism, version 9.2.0 (https://www.graphpad.com/scientifc-sofware/prism/). Statistical analyses were performed using Welch t-test for single-pair comparisons or one-way ANOVA with post hoc Tukey’s or Dunnett’s tests for multiple comparisons, where applicable.

## Supplementary Information

Supplementary Information.

## Data Availability

The datasets used and/or analyzed during the current study are available from the corresponding authors on reasonable request.
