# Supplementary material for: Oxidative stress monitoring in iPSC-derived motor neurons using genetically encoded biosensors of H2O2
Source: Sci Rep. 2022 May 27;12:8928. doi: 10.1038/s41598-022-12807-z (PMC9142597; doi:10.1038/s41598-022-12807-z)
Supplement: Supplementary file 1 — Supplementary Information. [file 41598_2022_12807_MOESM1_ESM.pdf]

Additional files to the paper

“Oxidative stress monitoring in iPSC-derived motor neurons using genetically encoded biosensors of H_2O_2”

Elizaveta I. Ustyantseva [1,*], Sophia V. Pavlova [1, 2, 3], Anastasia A. Malakhova [1, 2, 3], Kirill Ustyantsev [1], Suren M. Zakian [1, 2, 3], Sergey P. Medvedev [1, 2, 3,*]

* Correspondence: e.ustyantseva21@gmail.com, medvedev@bionet.nsc.ru
1 – The Federal Research Center Institute of Cytology and Genetics, Siberian Branch of Russian Academy of Sciences, 10, Lavrentiev Ave, 630090 Novosibirsk, Russia;

2 – Institute of Chemical Biology and Fundamental Medicine, Siberian Branch of the Russian Academy of Sciences, 8, Lavrentiev Ave., 630090 Novosibirsk, Russia;

3 – E. Meshalkin National medical research center of the Ministry of Health of the Russian Federation, 15 Rechkunovskaya Str., 630055 Novosibirsk, Russia;

Supplementary Table S1. List of primers and oligonucleotides used in the study

Name	Sequence, 5’-3’	Target
	Oligonucleotides	
AAVS1 crisprRNA	GUCACCAAUCCUGUCCCUAGGUU UUAGAGCUAUHCU	crisprRNA targeting *AAVS1*
SOD1-4 crisprRNA	GACUGCUGACAAAGAUGGUGGUU UUAGAGCUAUGCU	crisprRNA targeting exon 4 of *SOD1* (c.272A>C substitution)
SOD1-5 crisprRNA	GCAGAUGACUUGGGCAAAGGGUU UUAGAGCUAUGCU	crisprRNA targeting exon 5 of *SOD1* (c.382G>C substitution)
D91A-ssODN donor	A*G*A*TCACAGAATCTTCAATAGAC ACGTCGGCCACACCATCTTTGGCAG CAGTCACATTGCCCAAGTCTCCAAC ATGCCTAA TAATGAA*A*A*A	ssODN donor sequence (c.272A>C substitution in *SOD1*) * - Phosphorothioate bonds
G128R-ssODN donor	G*T*T*TCCTGTCTTTGTACTTTCTTCAT TTCCACCTTTGCGCAAGTCATCTGC TTTTTCATGAACCTGTAAAAAATTT TAAGAAGATAAC*T*T*T	ssODN donor sequence (c.382G>C substitution in *SOD1*) * - Phosphorothioate bonds
	DNA-probes for c.382G>C detection	
G128G-WT-FAM	FAM-CCTTTGCCCAAGTCATCTGC-BHQ1	Wild type allele detection
G128R-mut-VIC	VIC-CCTTTGCGCAAGTCATCTGC-BHQ2	c.382G>C mutant allele detection
	Primers	
SOD1-D91A-F	ATATCAGAGGCCTTGGGACATAG	Amplification of the exon 4 of *SOD1*
SOD1-D91A-R	TGAACTGCAAGTACAGTTTATCTGG	
SOD1-G128R-F	TGTCTTTGCAACACCAAGAAA	Amplification of the exon 5 of *SOD1*
SOD1-G128R-R	TTCACAGGCTTGAATGACAAA	
B2M-F	TAGCTGTGCTCGCGCTACT	Housekeeping reference gene for qPCR
B2M-R	TCTCTGCTGGATGACGTGAG	
RPL13-F	CCTGGAGGAGAAGAGGAAAGAGA	Housekeeping reference gene for qPCR
RPL13-R	TTGAGGACCTCTGTGTATTTGTCAA	
GAPDH-F	TGTTGCCATCAATGACCCCTT	Housekeeping reference gene for qPCR
GAPDH-R	CTCCACGACGTACTCAGCG	
HPRT1-F	GACTTTGCTTTCCTTGGTCAGG	Housekeeping reference gene for qPCR
HPRT1-R	AGTCTGGCTTATATCCAACACTTCG	
OCT4-F	CTTCTGCTTCAGGAGCTTGG	Pluripotency markers expression
OCT4-R	GAAGGAGAAGCTGGAGCAAA	
SOX2-F	GCTTAGCCTCGTCGATGAAC	
SOX2-R	AACCCCAAGATGCACAACTC	
NANOG-F	CAGCCCCGATTCTTCCACCAGTCCC	
NANOG-R	CGGAAGATTCCCAGTCGGGTTCACC	
HB9-F	GTCCACCGCGGGCATGATCC	Motor neuron markers expression
HB9-R	TCTTCACCTGGGTCTCGGTGAGC	
CHAT-F	GGAGGCGTGGAGCTCAGCGACACC	
CHAT-R	CGGGGAGCTCGCTGACGCAGTCTG	
ISL-F	AGCAGCCCAATGACAAAACT	
ISL-R	CTGAAAAATTGACCAGTTGCTG	
Myco-F	GGGAGCAAACAGGATTAGATACCCT	Mycoplasma

Myco-R	TGCACCATCTGTCACTCTGTTAACCTC	contamination detection
SOD1- qPCR-G128R-F	GTAGTGATTACTTGACAGCC	Amplification of the part of the exon 5 of *SOD1* for c.382G>C detection
SOD1- qPCR-G128R-R	CAATTACACCACAAGCCAA	
roGFP2-qPCR-F	TAGACGTTGTGGCAGTTGTAG	Biosensors expression
roGFP2-qPCR-R	GCTGAAGGGCATCGACTT	
rtTA-qPCR-F	GGACAGGCATCATACCCACTT	Transactivator expression
rtTA-qPCR-R	AGAGCACAGCGGAATGACTT	
HA_L-OUT	CCGGACCACTTTGAGCTCTAC	Target insertion of the biosensor/transactivator in *AAVS1*
Neo_in-R	GCCCAGTCATAGCCGAATAG	+ HA_L_OUT, target insertion of the transactivator
Puro_in-R	AGGCGCACCGTGGGCTTGTAC	+ HA_L_OUT, target insertion of the biosensor
M13-R	CAGGAAACAGCTATGAC	+Neo_in-R, off-target insertions of the AAVS1-Neo-M2rtTA donor
M13-F	GTAAAACGACGGCCAGT	+Puro_in-R, off-target insertions of the Cyto-roGFP2-Orp1/ Cyto-roGFP2-Orp1 donor
D91A-inner-F	CTTGGGCAATGTGACTGCCGA	c.272A>C detection in *SOD1* using Tetra-primer ARMS-PCR
D91A-inner-R	ATCGGCCACACCATCTTCGG	
D91A-outer-F	TGATGTTTAGTGGCATCAGCCCTAATC	
D91A-outer-R	GAAACCGCGACTAACAATCAAAGTGAA	
D91A-OT1-F	CAGGGTTTTGAGAAGATTAGATC	CRISPR SOD1-4 off-target site 1
D91A-OT1-R	TGTAGATGTAAAGGGCACCATAC	
D91A-OT2-F	TGCCAAGATATGCTTCAATGAAG	CRISPR SOD1-4 off-target site 2
D91A-OT2-R	TTCTTCCTCAGTAATCTTTAGCG	
D91A-OT3-F	AAATCCCTGTAGCCTCTTAGAAG	CRISPR SOD1-4 off-target site 3
D91A-OT3-R	CTTGCCTGTGGCTAGTTTATGTC	
D91A-OT4-F	TCACACAGCTTTCATCTACTAAG	CRISPR SOD1-4 off-target site 4
D91A-OT4-R	CCAAATCATGCAGCCATCAGATC	
D91A-OT5-F	GAGAAGAGACAAATCTTCAGTGC	CRISPR SOD1-4 off-target site 5
D91A-OT5-R	GTACATACTACTGCCATTTGGTC	
G128R-OT1-F	CAGCTAGCAGGATAACACATGTG	CRISPR SOD1-5 off-target site 1
G128R-OT1-R	AGCAGATGCCATGTTCATCAGTG	
G128R-OT2-F	AAGTGACCAGTCAATGTTAGCTG	CRISPR SOD1-5 off-target site 2
G128R-OT2-R	CCTTTCAGGTAACAGGAAGCTAC	
G128R-OT3-F	ACAAAGGTGGAATTCCAAGTTAG	CRISPR SOD1-5 off-target site 3
G128R-OT3-R	TACAAAGTGATCTTGACAGGTAC	
G128R-OT4-F	GGCATCTTGAATGGCATCAGCTG	CRISPR SOD1-5 off-target site 4
G128R-OT4-R	AGAATACACTCTACTTCTTGACC	
G128R-OT5-F	GCAAGGGAGAGAGTGACTTGATC	CRISPR SOD1-5 off-target site 5
G128R-OT5-R	GAACCACTGCTCTGAAAGATTAC	

Supplementary Table S2. List of iPSC lines used in the study

Name	Genotype	Generation and characteristics	HpscReg #	Remarks
K7-4Lf	*SOD1*$^{WT/WT}$	(Malakhova et al., 2020)	ICGi022-A	Exome sequencing data: https://www.ncbi.nlm.nih.gov/sra/?term=SRR11413027; isogenic control to the ICGi022-A-1 and ICGi022-A-2
iALS	*SOD1*$^{D91A/D91A}$	(Ustyantseva et al., 2020)	ICGi04-A	
SOD1-D91A	*SOD1*$^{D91A/\Delta105}$	Present study	ICGi022-A-1	Genetically modified subclone of ICGi-022-A. Both alleles of the *SOD1* gene were modified by CRISPR/Cas9 with [c.272A>C] + [c.238+675_281del] mutations.
SOD1-G128R	*SOD1*$^{G128R/K129X}$	Present study	ICGi022-A-2	Genetically modified subclone of ICGi022-A. Both alleles of the *SOD1* gene were modified by CRISPR/Cas9 with [c.382G>C] + [c.385_386 delinsTG] mutations, leading to the G128R and K129* substitutions in the protein sequence, respectively.
K7-4Lf-Cyto1, K7-4Lf-Cyto2, K7-4Lf-Cyto3	*SOD1*$^{WT/WT}$	Present study	-	Contain transgenes of the cytosolic H_2O_2 biosensor Cyto-roGFP2-Orp1 and tetracycline-transactivator (rtTA) in *AAVS1* locus
K7-4Lf-Mito1, K7-4Lf-Mito2, K7-4Lf-Mito3	*SOD1*$^{WT/WT}$	Present study	-	Contain transgenes of the mitochondrial H_2O_2 biosensor Mito-roGFP2-Orp1 and tetracycline-transactivator (rtTA) in *AAVS1* locus
iALS-Cyto1, iALS-Cyto2, iALS-Cyto3	*SOD1*$^{D91A/D91A}$	Present study	-	Contain transgenes of the cytosolic H_2O_2 biosensor Cyto-roGFP2-Orp1 and tetracycline-transactivator (rtTA) in *AAVS1* locus
iALS-Mito1, iALS-Mito2, iALS-Mito3	*SOD1*$^{D91A/D91A}$	Present study	-	Contain transgenes of the mitochondrial H_2O_2 biosensor Mito-roGFP2-Orp1 and tetracycline-transactivator (rtTA) in *AAVS1* locus
SOD1-D91A-Cyto1, SOD1-D91A-Cyto2, SOD1-	*SOD1*$^{D91A/\Delta105}$	Present study	-	Contain transgenes of the cytosolic H_2O_2 biosensor Cyto-roGFP2-Orp1 and tetracycline-transactivator (rtTA) in *AAVS1* locus

D91A-Cyto3				
SOD1-D91A-Mito1, SOD1-D91A-Mito2, SOD1-D91A-Mito3	*SOD1*$^{D91A/\Delta105}$	Present study	-	Contain transgenes of the mitochondrial H_2O_2 biosensor Mito-roGFP2-Orp1 and tetracycline-transactivator (rtTA) in *AAVS1* locus
SOD1-G128R-Cyto1, SOD1-G128R-Cyto2, SOD1-G128R-Cyto3	*SOD1*$^{G128R/K129X}$	Present study	-	Contain transgenes of the cytosolic H_2O_2 biosensor Cyto-roGFP2-Orp1 and tetracycline-transactivator (rtTA) in *AAVS1* locus
SOD1-G128R-Mito1, SOD1-G128R-Mito2, SOD1-G128R-Mito3	*SOD1*$^{G128R/K129X}$	Present study	-	Contain transgenes of the mitochondrial H_2O_2 biosensor Mito-roGFP2-Orp1 and tetracycline-transactivator (rtTA) in *AAVS1* locus

Supplementary figure S3

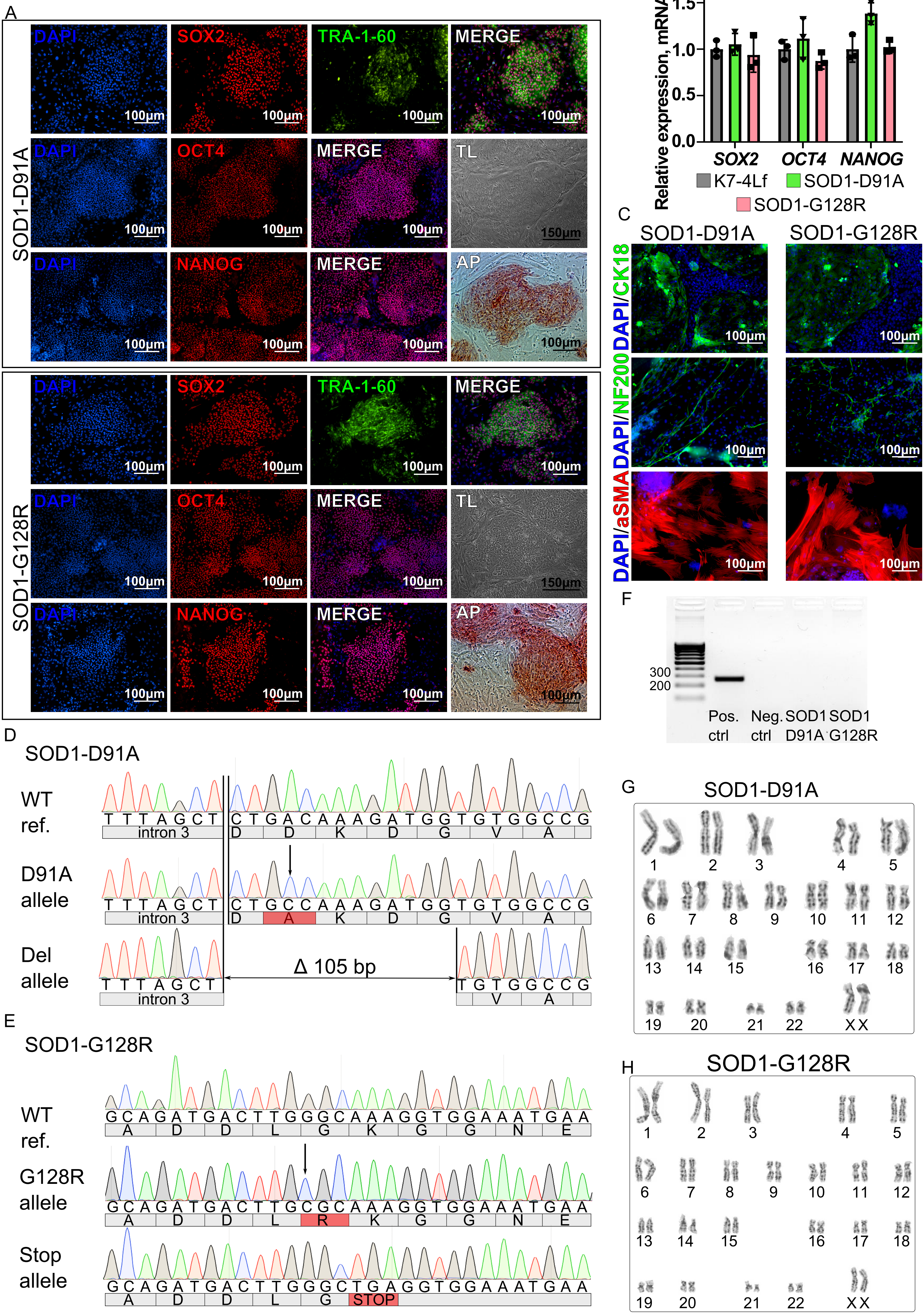

Supplementary Figure S3: SOD1-D91A and SOD1-G128R iPSC lines characterization. **A** Pluripotent features of the SOD1-D91A and SOD1-G128R iPSC lines. Immunofluorescent staining for transcriptional factors: SOX2, OCT4, NANOG, and surface antigen TRA-1-60. Nuclei are visualized with DAPI. Scale bar 100 μM. IPSC colony morphology (TL – transmitted light), scale bar 150 μM. Alkaline phosphatase (AP) staining, scale bar 100 μM. **B** RT-qPCR analysis of mRNA expression of *SOX2*, *OCT4*, and *NANOG* in SOD1-D91A and SOD1-G128R iPSCs. Data (N = 3 RNA samples for each genotype) are normalized to the parental iPSC line (K7-4Lf) and presented as the mean ± standard deviation. **C** Immunofluorescent staining of the products of *in vitro* spontaneous differentiation for endodermal (CK18), ectodermal (NF200), and mesodermal (αSMA) markers. Nuclei are visualized with DAPI. Scale bar 100 μM. **D** Partial sequence of exon 4 of the *SOD1* gene of SOD1-D91A with the corresponding amino acid sequence. WT ref – reference sequence (parental iPSC line K7-4Lf); D91A allele– allele with the c.272A>C single nucleotide substitution; Del allele – allele with 105-nucleotide deletion. **E**. Partial sequence of exon 5 of the *SOD1* gene of SOD1-G128R with the corresponding amino acid sequences. WT ref. – reference sequence (parental iPSC line K7-4Lf); G128R allele – allele with the c.382G>C single nucleotide substitution; Stop allele – allele with premature termination codon. **F** Mycoplasma contamination detection with specific primers. Neg. ctrl – H_2O, Pos. ctrl – mycoplasma contaminated cell line. Karyotyping of the SOD1-D91A (**G**) iPSC line and SOD1-G128R (**H**) iPSC lines.

Supplementary Figure S4

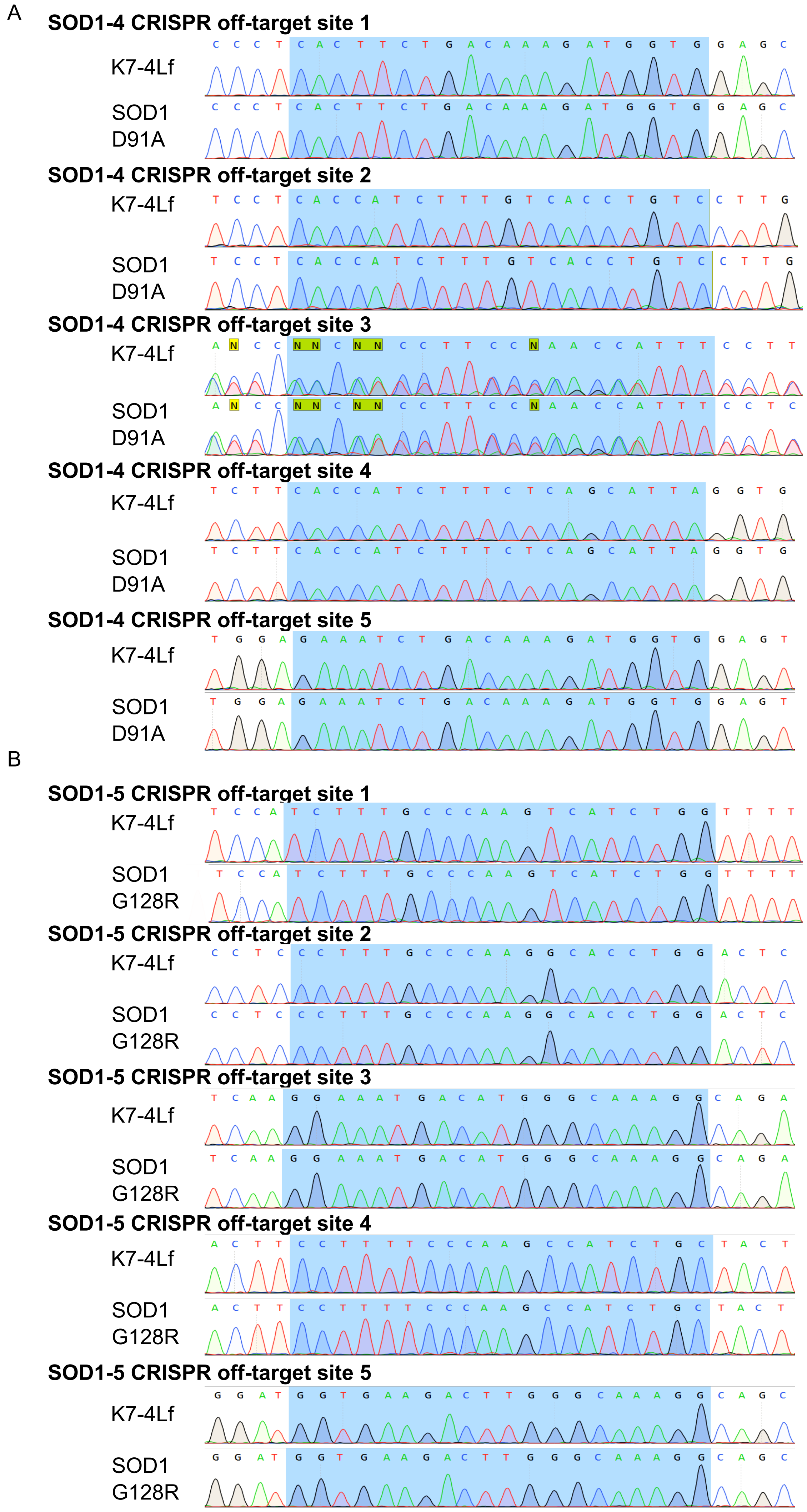

Supplementary Figure S4: Partial sequences of the top 5 off-target sites predicted for CRISPR SOD1-4 and SOD1-5 used in the study. **A** Genomic DNA of SOD1-D91A iPSC line. **B** Genomic DNA of SOD1-G128R iPSC line. Genomic DNA of the parental K7-4Lf iPSC line was used as a control. Potential off-target protospacers are underlined with the blue color.

Supplementary figure S5.

A

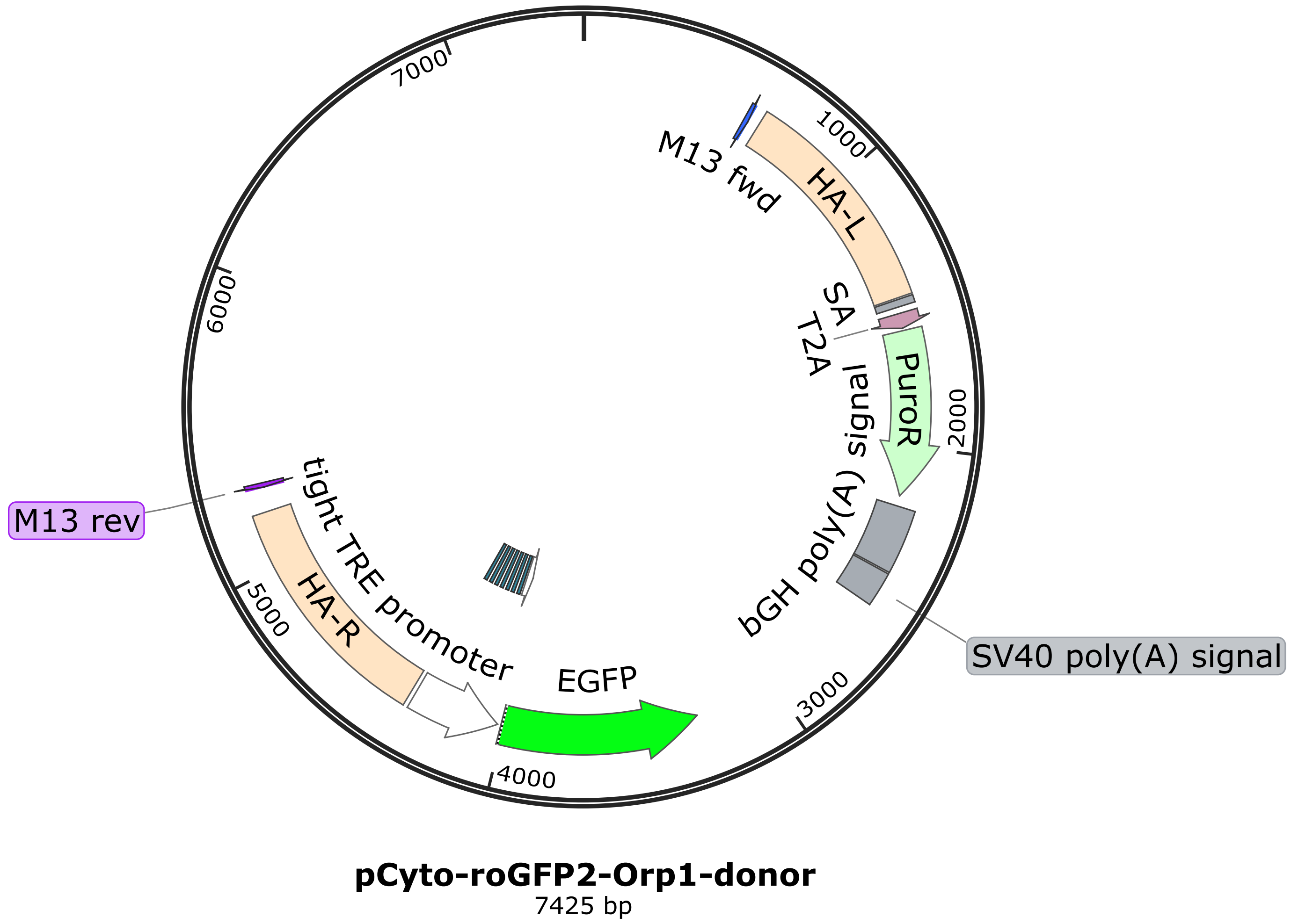

A

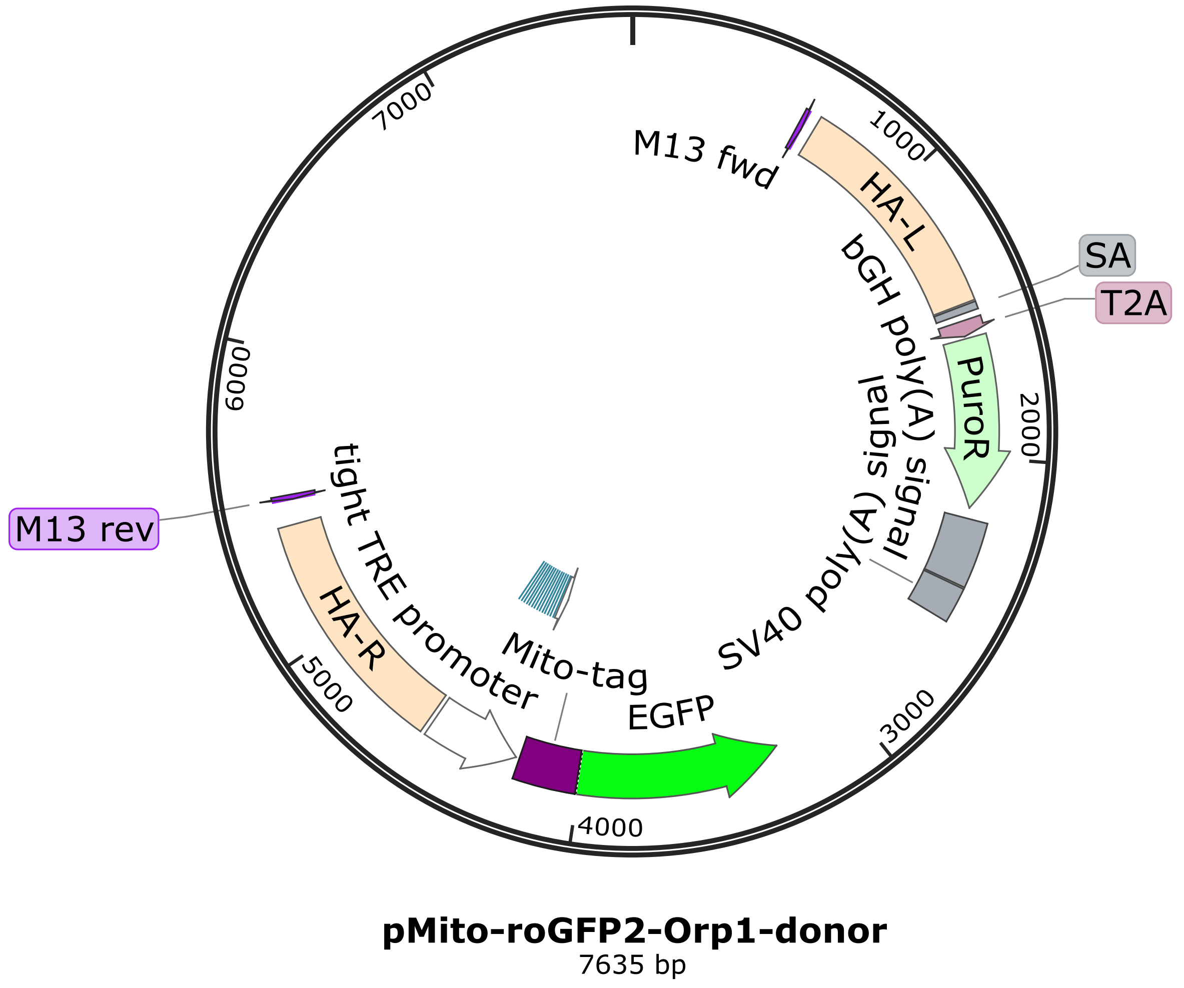

Supplementary Figure S5: pCyto-roGFP2-Orp1-donor (**A**) and pMito-roGFP2-Orp1-donor (**B**) plasmid maps. Schematic maps of the donor plasmids used for CRISPR/Cas9 target insertion of the biosensors in the *AAVS1* locus. Basic elements: homologous arms, puromycin resistance gene, promoters, Tet-On elements for doxycycline-controllable expression, and biosensors' functional elements are present. All maps were constructed with SnapGene®.

Supplementary figure S6

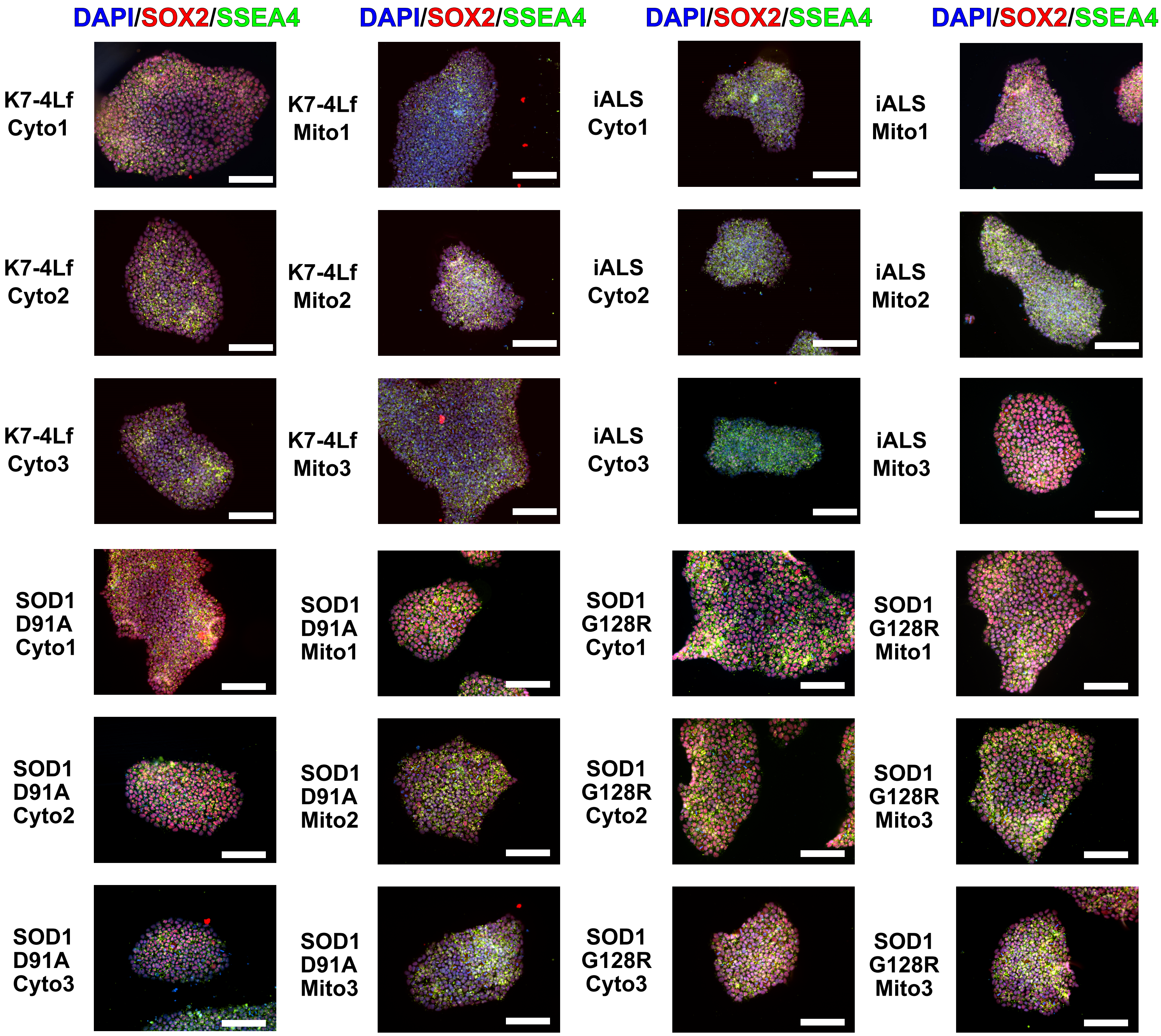

iPSC line	Cyto-roGFP2-Orp1	Mito-roGFP2-Orp1
K7-4Lf	5 clones	3 clones
SOD1-D91A	14 clones	11 clones
SOD1-G128R	12 clones	12 clones
iALS	17 clones	11 clones

Supplementary Figure S6: SOX2 and SSEA4 staining of the transgenic iPSC clones expressing Cyto-roGFP2-Orp1 (_Cyto) or Mito-roGFP2-Orp1 (_Mito) biosensors. Nuclei are visualized with DAPI, scale bar 100 μM. At the bottom is a table with the number of clones with respective insertions, obtained in the study.

Supplementary figure S7

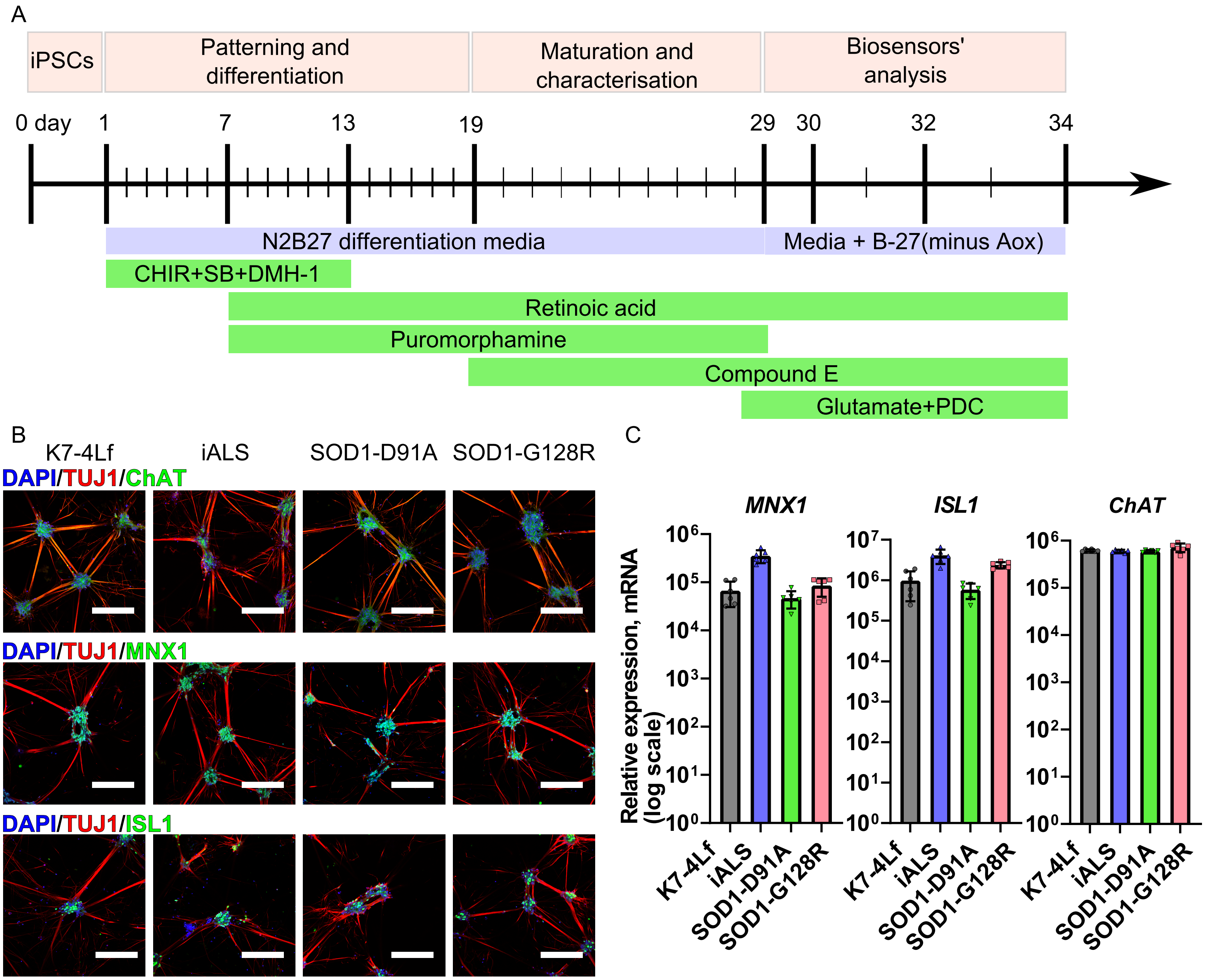

Supplementary Figure S7: Generation of iPSC-derived motor neurons expressing the Cyto-roGFP2-Orp1 and Mito-roGFP2-Orp1 biosensors. **A** Schematic of the differentiation protocol and a timeline for MN characterization and analysis. The main stages of the protocol are indicated above the timeline. The medium composition (basal medium and small-molecule inhibitors) for every stage is indicated below the timeline. The concentrations of all the compounds shown in the figure are present in the Materials and Methods section. **B** Representative images of the K7-4Lf, iALS, SOD1-D91A, and SOD1-G128R MNs positively stained for MN markers: ChAT, MNX1, and ISL1 on the differentiation day 20. Nuclei are visualized with DAPI, scale bar 100 µm. **C** RT-qPCR analysis of mRNA expression of *MNX1*, *ISL1*, and *ChAT* in K7-4Lf, iALS, SOD1-D91A, and SOD1-G128R MNs on the differentiation day 29. Data (N = 6 independent differentiations) are normalized to the expression in the K7-4Lf iPSC line and presented as the mean ± standard deviation (S.D.).

Supplementary figure S8

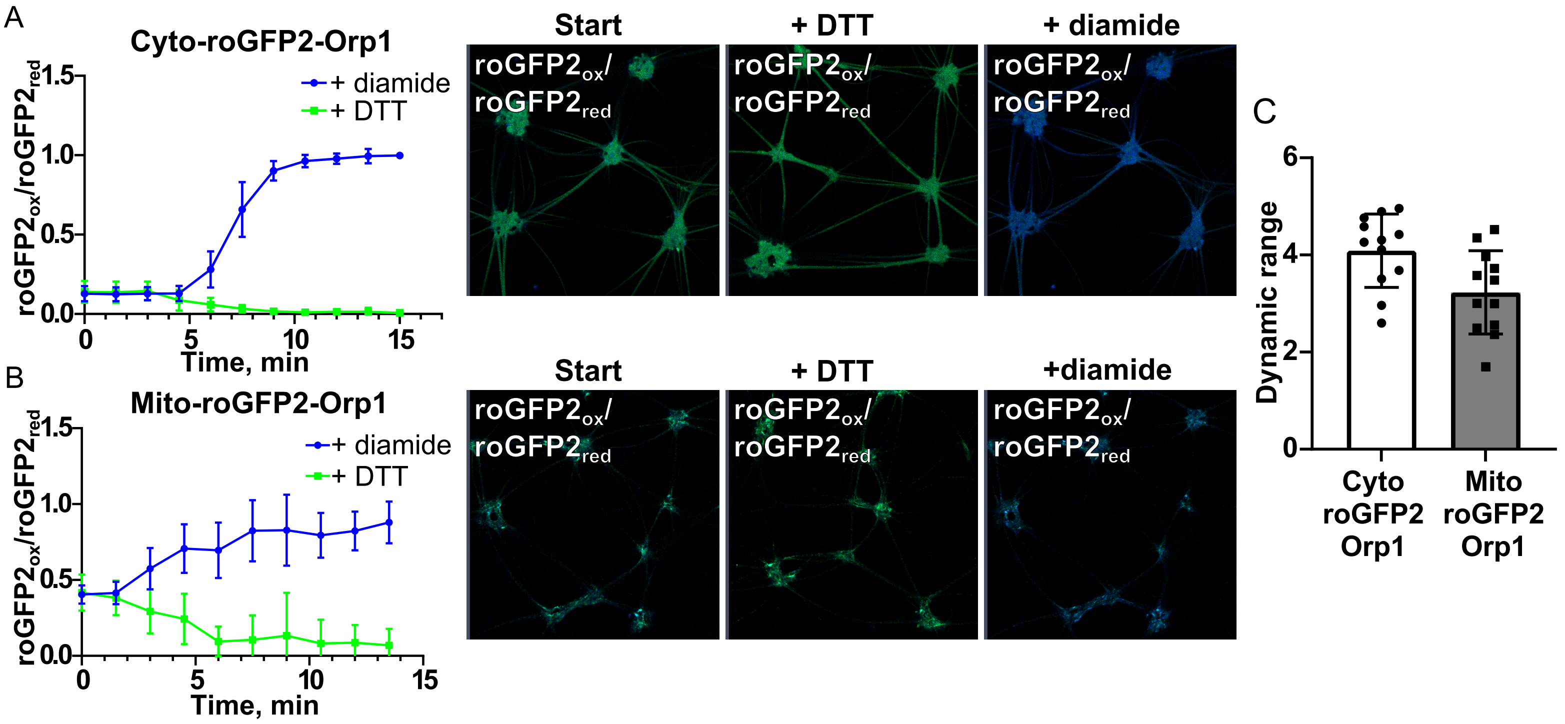

Supplementary Figure S8: Evaluation of the dynamic range of Cyto-roGFP2-Orp1 and Mito-roGFP2-Orp1 in iPSC-derived motor neurons (MNs). The reaction of the Cyto-roGFP2-Orp1-expressing (**A**) and Mito-roGFP2-Orp1-expressing (**B**) MNs to the diamide and DTT addition with the representative images of the intact MNs (Start), fully reduced (+ DTT) and fully oxidized (+ diamide) MNs. Data (N = 9 independent experiments) are normalized to the maximum oxidation and reduction values and presented as the mean ± standard deviation (S.D.). The Images present an overlap of the 405 nm (oxidized roGFP2) and 488 nm (reduced roGFP2) channels colored in blue and green pseudocolors, respectively. **C** Dynamic range of the Cyto-roGFP2-Orp1 and Mito-roGFP2-Orp1 biosensors expressed in MNs. Data (N = 12 independent experiments) are the mean ± S.D.

Supplementary figure S9

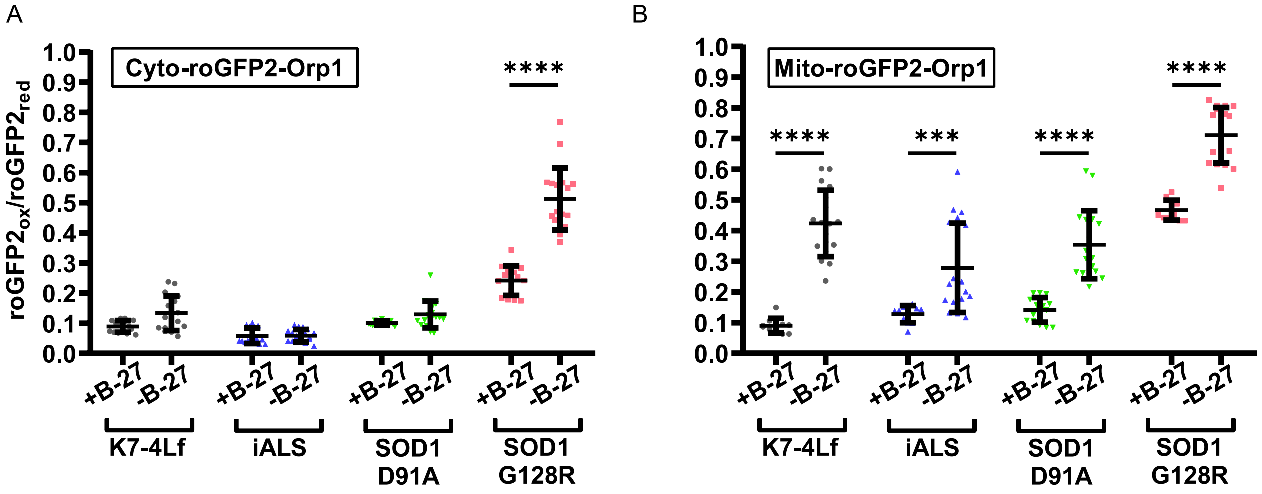

Supplementary Figure S9: Basal levels of H_2O_2 in the cytoplasm (Cyto-roGFP2-Orp1, **A**) and mitochondria (Mito-roGFP2-Orp1, **B**) measured on the differentiation day 29 in the biosensor-expressing motor neurons (MNs). The medium was either kept standard (+ B-27) or replaced 24 hours before the measurement for nutrient-deficient medium (-B-27). Values were obtained by analyzing the microscopic images of the respective motor neurons. Data (N = 10-15, and 18 for B-27 deprived samples, fields of view, collected from MNs, derived from three iPSC lines with the same genotype) on the graphs are regrouped from the Fig 4A and 4D and present as mean ± standard deviation. **** $p < 0.0001$, non-significant data are not presented. Welch t-test was used for paired comparisons.

Supplementary Figure S10

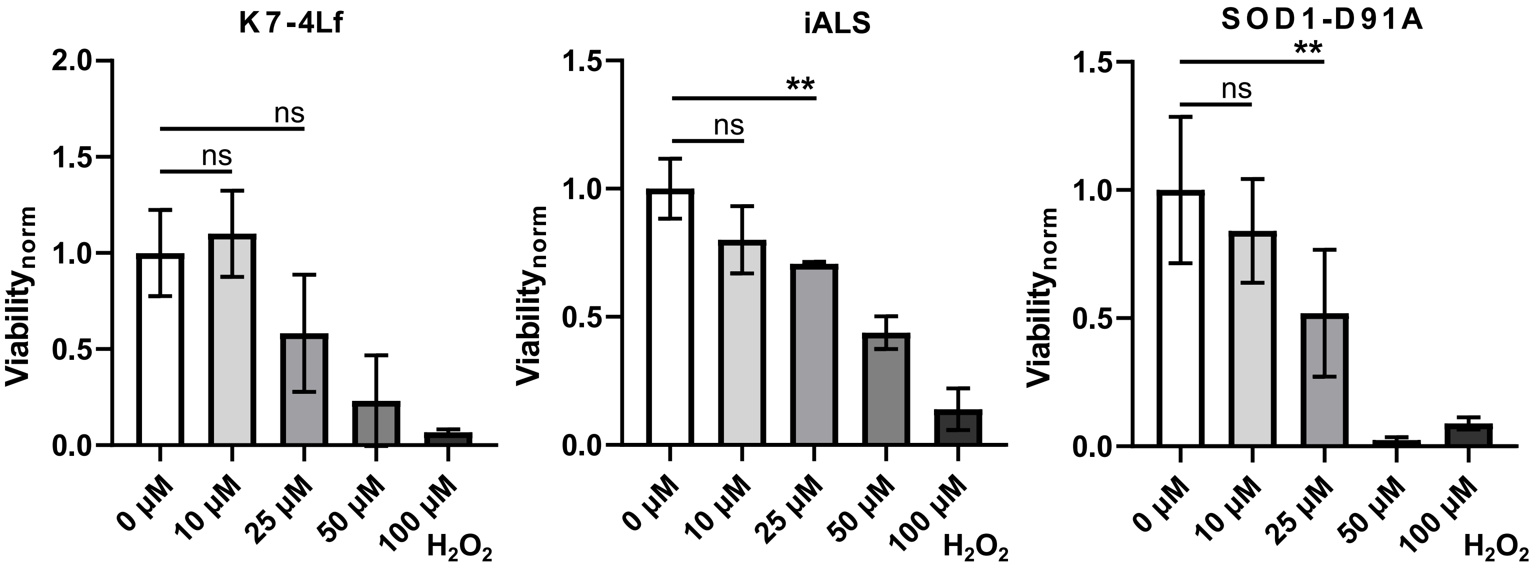

Supplementary Figure S10: Viability of K7-4Lf, iALS, and SOD1-D91A motor neurons after treatment with 10 μM, 25 μM, 50 μM, and 100 μM H_2O_2, determined by XTT-assay. Data (N=3 measurements) are normalized to the untreated (0 μM) sample and presented as the mean ± S.D.

Supplementary Table S11. List of the antibodies used in the study

Antibody	Vendor	Cat #	Host	Dilution
**Primary antibody**				
Pluripotency markers				
OCT4	BD Transduction Lab	611202	Mouse monoclonal	1:200
NANOG	Abcam	ab62734	Rabbit polyclonal	1:200
SOX2	Cell Signaling	3579	Rabbit polyclonal	1:400
SSEA4	Abcam	ab16287	Mouse monoclonal	1:50
TRA-1-60	Abcam	ab16288	Mouse monoclonal	1:200
Ecto-, meso-, endoderm markers				
NF200	Sigma	N4142	Rabbit polyclonal	1:500
aSMA	Dako		Mouse monoclonal IgG2a	
CK18	EMD Millipore	MAB3236	Mouse monoclonal IgG1	1:200
Motor neuron markers				
TUJ1	Biolegend	801213	Mouse monoclonal IgG2	1:1000
HB9	EMD Millipore	ABN174	Rabbit polyclonal	1:1000
ISL1	Abcam	ab109517	Rabbit polyclonal	1:500 (1:100 flow cytometry)
ChAT	EMD Millipore	AB144P	Rabbit polyclonal	1:100
**Secondary antibody**				
Alexa Fluor 488 goat anti-rabbit IgG (H+L)	Thermo Fisher Scientific	A11008	Goat polyclonal	1:400
Alexa Fluor 568 goat anti-rabbit IgG (H+L)	Thermo Fisher Scientific	A11011	Goat polyclonal	1:400 (1:2000 flow cytometry)
Alexa Fluor 488 goat anti-mouse IgG (H+L)	Thermo Fisher Scientific	A11029	Goat polyclonal	1:400
Alexa Fluor 568 goat anti-mouse IgG (H+L)	Thermo Fisher Scientific	A11004	Goat polyclonal	1:400
Alexa Fluor 568 goat anti-mouse IgM	Thermo Fisher Scientific	A21043	Goat polyclonal	1:400
Alexa Fluor 488 rabbit anti-goat IgG (H+L)	Thermo Fisher Scientific	A11078	Goat polyclonal	1:400

Supplementary figure S12

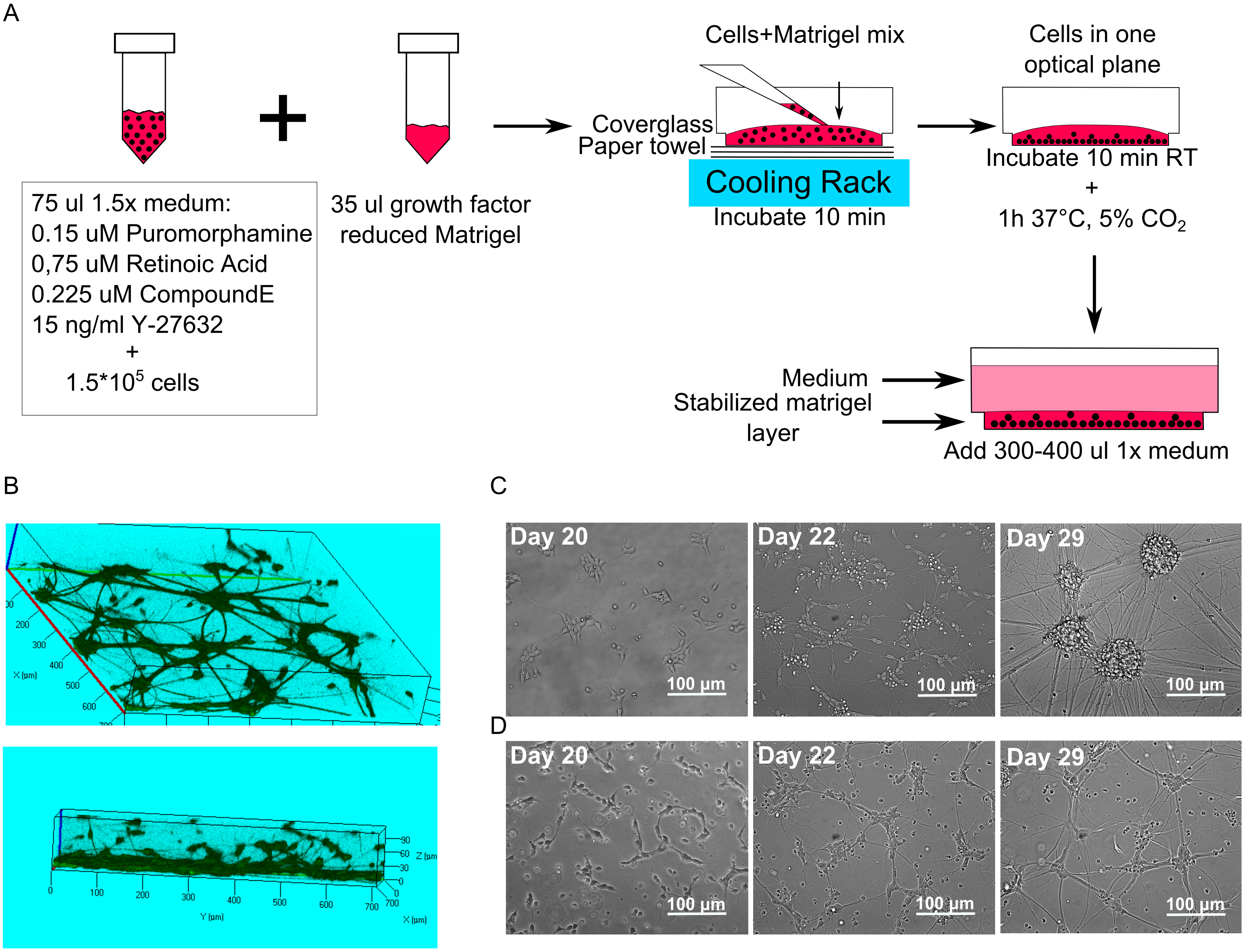

Supplementary Figure S12: Graphical description of the microscopy sample preparation for live MN imaging. **A** Schematic representation of the protocol for MN seeding. **B** 3D reconstruction of the cell layer (differentiation day 29) obtained with the protocol. Morphology of MNs seeded for the maturation on the top of the Matrigel (**C**) or inside of 33% Matrigel layer (**D**) at the indicated days of differentiation.

Supplementary methods

Preparation of live motor neuron samples for microscopy

Motor neurons have a low surface attachment, making long-term microscopy experiments difficult. Therefore, in the biosensors experiments, the cells were seeded for maturation on the cell imaging coverglasses inside a layer of 33% Matrigel. To do so, we resuspended the cells in the 1.5× cold NDM (supplemented with 15 ng/ml Y-27632, 0.75 µM retinoic acid, and 0.225 µM Compound E), making a suspension with 1.5×10^5 cells/75 µl (1.5×10^5 cells/well). Then, we mixed 75 µl of the cell suspension with 35 µl of growth factor reduced Matrigel (Corning) and quickly applied 100 µl of the mix on the surface of the chilled cell imaging coverglass, standing on a cold tube cooling rack turned upside down and covered with a paper towel. Using the tip of a pipette, we carefully spread the mix over the surface and left it on the cooling rack for 10 minutes to let the cells fall to the bottom before the Matrigel polymerized. Next, we transferred the coverglasses on top of the working surface and left them for another 10 minutes, and then, the coverglasses were carried over to a CO_2 incubator for 1h for Matrigel stabilization. After 1 hour, we added 300-400 µl of the fourth step medium supplemented with Y-27632 (10 ng/ml) on top of the stabilized Matrigel layer **(Supplementary Fig. S11)**.

Image acquisition

Cells and solutions preparation

To measure H_2O_2 utilization in real-time, we replaced the standard neuronal culture medium with the deficit medium (F12/DMEM:Neurobasal – 50:50, 1x N2 supplement, 2 µg/ml doxycycline hyclate) the day before the experiment, unless otherwise indicated. 1 hour before the experiment the medium was removed from the analyzed wells (so as to not disturb the Matrigel layer with neurons inside), and replaced with warm HBSS + Ca^{2+}, Mg^{2+}. The cells were incubated in a CO_2 incubator to remove the residual medium components from the Matrigel layer. Then, the old HBSS was almost entirely removed (with a residue of ~ 50 µl/well) and replaced with the fresh HBSS (~ 300 µl/well). The final volume of the sample in one well is 450 µl: 300+50 µl of HBSS, 100 µl of the Matrigel layer with MNs.

The stock and working solutions were prepared freshly on the day of the experiment. The water stock solutions of 1 M DTT (Sigma-Aldrich) and 0.2 M diamide (Sigma-Aldrich) were made from a powder; 10 mM H_2O_2 stock solution was made from hydrogen peroxide solution (30% w/w, Sigma-Aldrich).

From these stock solutions, the working solutions were prepared:

1) 10 µl l 1M DTT + 190 µl HBSS (final concentration 50 µM);
2) 5 µl 0.2M diamide +195 µl HBSS (final concentration 5 µM);
3) 2 µl 10 mM H_2O_2 + 198 µl HBSS (final concentration 100 µM).

Microscopy settings

We used a confocal Zeiss LSM-780 laser scanning microscope (Pan-Apochromat 20× objective) equipped with the 488 nm argon laser, the 405 nm UV diode laser, and a climate chamber connected with the temperature and CO_2 control modules. Cell imaging coverglass with the cells was placed on the stage without a lid, covered with a CO_2 cover, and left for 5-10 minutes for temperature equilibration. Tubes with the working solutions were also put inside the climate chamber. The cells were visualized with transmitted light during the equilibration to achieve a stable focus. Actual microscopy settings varied between the samples and required customization for obtaining a quality image. Table 1 describes the initial microscopy setup. Images did not contain oversaturated pixels and had a high noise-to-signal ratio.

Table 1. Initial microscopy setup (Zeiss LSM-780) for imaging of Cyto-roGFP2-Orp1 and Mito-roGFP2-Orp1 expressing motor neurons

**Mode**	**Channel mode**
Channels:	

Switch track every	«Frame»
Track 1	405 nm (DAPI, roGFP2ox) + T-PMT (Transmission)
Track 2	488 nm (EGFP, roGFP2red)
Light Path:	
Track 1+ Track 2	500-530 nm
Acquisition mode:	
Scan mode	Frame
Frame size	512x512 (1024x1024 for photo)
Scanning speed (pixel dwell)	9 (1,58 μsec)
Averaging	1
Bit depth	16 bit
Zoom	0.6
Channel/Laser settings:	
405-nm line	
Attenuator (transmission)	5%
Pinhole	74.5
Gain	800
Digital gain	1
488-nm line	
Attenuator (transmission)	2%
Pinhole	74.5
Gain	800 – Cyto-roGFP2-Orp1, 700 - Mito-roGFP2-Orp1
Digital gain	1

Biosensor calibration using DTT/diamide

To determine the states of the maximum oxidation and reduction possible for the biosensors, we treated the cells with diamide and DTT, respectively, using the following procedure:

1) In the mode "Position" we set up three fields of view in each of the two adjacent wells and saved their coordinates;
2) Next, we set the microscope to the "time series" mode with 18 cycles of 90 s;
3) Then, we started the time-series experiment and paused it after cycle 3, added 50 μl of the warm DTT working solution into one well (final concentration 5 μM) and 50 μl of the warm diamide working solution into the other well (final concentration 0.5 μM), trying not to disturb the coverglass, and resumed the time series;
4) We waited for the cells to become fully reduced/oxidized (usually 8-10 minutes): the signal in each sample reached the plateau. In the DTT well intensity of the 488 nm signal slightly increased, while the intensity of the 405 nm signal slightly decreased; in the diamide well intensity of the 488 nm signal intensity considerably decreased, while the intensity of the 405 nm signal considerably increased.

The calibration procedure was performed for every sample before the other experiments, and the values of the maximum oxidation/reduction were used for the dynamic range calculation and data normalization.

Basal H_2O_2 level measurement and measurement of H_2O_2 utilization in real-time.

To measure the Cyto-roGFP2-Orp1 and Mito-roGFP2-Orp1 signals in motor neurons, we applied the microscope settings determined during the calibration. To measure basal H_2O_2 levels in the cytoplasm and mitochondria, we obtained images from MNs derived from the K7-4Lf, SOD1-D91A, SOD1-G128R, and iALS iPSC lines. To measure H_2O_2 utilization in real-time, we applied the same approach as for calibration, but with 33 cycles, each 3 minutes long. We

prepared the cells as described, mounted the coverglasses on the stage, and equilibrated the temperature in the climate chamber. We added 50 µl of the warm working H_2O_2 solution (final concentration 10 µM) to the cells after 2nd-3rd cycle, and continued the time series for another 30 cycles (1.5 hours).

Data normalization

The images were saved as 16-bit .tiff files and processed by ImageJ. For the analysis, the images were converted to the 32-bit format. Single pictures were split to the 405 nm ($roGFP2_{ox}$), 488 nm ($roGFP2_{red}$), and transmitted light channels. The intensity of the 405 and 488 channels was thresholded, and values below the threshold were set to "not a number" (NaN). A ratio image was created by dividing the 405 nm image by the 488 nm image ($roGFP2_{ox}/roGFP2_{red}$), and the mean intensity of the resulting image was measured. The time-series images were processed similarly. The files were imported in ImageJ as stacks, converted to the 32-bit format, and split using the "Stacks-Shuffling-Deinterleave" plugin. The threshold was then adjusted for both channels, and the values below the threshold were set to "not a number" (NaN). A resulting ratio of images was created by dividing the 405 nm image by the 488 nm and measured. The $roGFP2_{ox}/roGFP2_{red}$ ratios of the single images were used to describe the basal level of H_2O_2. The $roGFP2_{ox}/roGFP2_{red}$ ratios obtained from the time series were used for measurement of H_2O_2 utilization in real-time. For each cell type, data were normalized to a fraction of the maximum oxidation/reduction state obtained during calibration, according to the formula (1):

(1)

$$\text{Normalized}\ \frac{\text{roGFP2ox}}{\text{roGFP2red}} = \left. \text{Sample}\frac{\text{roGFP2ox}}{\text{roGFP2red}} - \text{DTT}\frac{\text{roGFP2ox}}{\text{roGFP2red}} \middle/ \text{Diamide}\frac{\text{roGFP2ox}}{\text{roGFP2red}} - \text{DTT}\frac{\text{roGFP2ox}}{\text{roGFP2red}} \right.$$

The normalized data were used for visualization, comparison, and statistical analysis. To automate the image analysis, we wrote a custom imageJ macro script (available upon request).

H_2O_2 utilization analysis

The H_2O_2 utilization process was characterized by the maximum changes of the biosensor's oxidation (max delta) and recovery rate after H_2O_2 addition. The max delta of the biosensor's oxidation was calculated by subtracting the initial normalized value of the roGFP2ox/roGFP2red ratio recorded at the "0" time point from the normalized maximum oxidation value of the roGFP2ox/roGFP2red ratio obtained after H_2O_2 addition (usually after 30 minutes of recording). The recovery rate was calculated by subtracting the final normalized value of the roGFP2ox/roGFP2red ratio recorded at the end of the time series from the normalized maximum oxidation value with subsequent division of the resulting numbers to the time interval between these two time points (in hours).
